# Bcl-x_L_ inhibition enhances Dinaciclib-induced cell death in soft-tissue sarcomas

**DOI:** 10.1038/s41598-019-40106-7

**Published:** 2019-03-07

**Authors:** Santi Rello-Varona, Miriam Fuentes-Guirado, Roser López-Alemany, Aida Contreras-Pérez, Núria Mulet-Margalef, Silvia García-Monclús, Oscar M. Tirado, Xavier García del Muro

**Affiliations:** 1 Sarcoma Research Group, Oncobell Program, Bellvitge Biomedical Research Institute (IDIBELL), L’Hospitalet de Llobregat, Barcelona, Spain; 2 Sarcoma Multidisciplinary Unit, Institut Català d’Oncologia-ICO, L’Hospitalet de Llobregat, Barcelona, Spain; 3 CIBERONC, Carlos III Institute of Health (ISCIII), Madrid, Spain; 4 Clinical Sciences Department, School of Medicine, Universitat de Barcelona, Barcelona, Spain

## Abstract

Soft-tissue sarcomas (STS) are an uncommon and heterogeneous group of malignancies that result in high mortality. Metastatic STS have very bad prognosis due to the lack of effective treatments. Dinaciclib is a model drug for the family of CDK inhibitors. Its main targets are cell cycle regulator CDK1 and protein synthesis controller CDK9. We present data supporting Dinaciclib ability to inactivate *in vitro* different STS models at nanomolar concentrations. Moreover, the different rhythms of cell death induction allow us to further study into the mechanism of action of the drug. Cell death was found to respond to the mitochondrial pathway of apoptosis. Anti-apoptotic Bcl-x_L_ was identified as the key regulator of this process. Already natural low levels of pro-apoptotic proteins BIM and PUMA in tolerant cell lines were insufficient to inhibit Bcl-x_L_ as this anti-apoptotic protein showed a slow decay curve after Dinaciclib-induced protein synthesis disruption. Combination of Dinaciclib with BH3-mimetics led to quick and massive apoptosis induction *in vitro*, but *in vivo* assessment was prevented due to liver toxicity. Additionally, Bcl-x_L_ inhibitor A-1331852 also synergized with conventional chemotherapy drugs as Gemcitabine. Thus, Bcl-x_L_ targeted therapy arises as a major opportunity to the treatment of STS.

## Introduction

Soft-tissue sarcomas (STS) are a group of tumors derived from mesenchymal precursors with scarce incidence and rich variability^1^. Tumors arising from non-epithelial extra-skeletal tissue are generally accounted as STS^2^. There has been much improvement in the understanding of the drivers of STS entities: (i) STSs driven by specific chromosome fusions leading to generation of anomalous transcription factors (like *FUS-CHOP* in myxoid liposarcoma) or chromatin remodelers (*SS18-SSX* in synovial sarcoma); (ii) STS that rely on specific mutations (*KIT* in gastrointestinal stromal tumors) and (iii) other STS driven by more complex genomic rearrangements (like leiomyosarcomas or some fibrosarcomas)^3,4^.

STS incidence is difficult to estimate due to their variability, and some reports claim that the usual figures could be underestimations^1,5^. Clinical prognosis and therapeutic outcome is also highly variable in STS^2^. When it is possible, the complete clinical resection make full recovery achievable. However, almost half of the patients will develop metastatic disease. Five-year survival rates are still below 50%. So, the weight of STS in total cancer death toll is clearly disproportionate to its incidence^4,6^.

Thus, STS can benefit for new therapeutic approaches^6^. Among the molecular targeted drugs in development, the group of Cyclin-Dependent Kinases (CDKs) inhibitors is one of those concealing major interest^7^. CDKs constitute a wide family of Ser/Thr protein kinases that require binding with cyclins to act. This coupling enables a complex panorama of interactions that keep track on the activation/suppression of specific pathways during cell cycle^8^. Several CDK inhibitors have been identified and tested *in vitro* as anti-cancer agents^7,9^. The initial aim of CDK inhibitor strategy was the disruption of cell cycle sequence-of-events in order to induce cell death^9,10^. But it was soon understood that CDKs exert more powerful effects over other processes of cell physiology like transcription regulation, RNA splicing or protein folding^9^.

Dinaciclib is a promising CDK inhibitor, extensively proved pre-clinically^11^. Its known affinities encompass CDK1 (IC_50_ = 3 nM), CDK2 (IC_50_ = 1 nM), CDK5 (IC_50_ = 1 nM) and CDK9 (IC_50_ = 4 nM)^12^. Most studies concerning Dinaciclib activity have been focused on the CDK1 control of mitotic entry and CDK9 regulation of gene transcription^13–15^. CDK9-dependent down regulation of anti-apoptotic Bcl-2 family member Mcl-1 is commonly regarded as the main mechanism of action of this drug^16,17^. Some Phase I clinical trials (mostly in pediatric leukemia) have also been performed with Dinaciclib. Anti-cancer activity was found to be encouraging, but not sufficient for planning monotherapy treatments. Further use of Dinaciclib is thought to rely on combination therapies^13,18,19^.

Combination therapies constitute a hot spot in oncology research. It has become clear its benefits avoiding tumor evolution in favor of drug resistant phenotypes^20^. Moreover, combination therapies work better than monotherapy even in the absence of synergistic behavior^21^. BH3-mimetics are a new class of anti-cancer drugs particularly interesting for these combinations. They are aimed to disturb the balance of the different proteins of the Bcl-2 family, thus favoring apoptosis triggering^22,23^. Alone, BH3-mimetics have been successfully used in chronic lymphocytic leukemia since the FDA approval of Venetoclax^24^. BH3-mimetics work better when the cells are already undergoing an apoptotic signaling process that has been compensated by expression or activity changes in the Bcl-2 family of proteins. Cells became addicted to these compensatory mechanisms creating then an Achilles’ heel for cancer cells^23^. Recent screenings are showing that BH3-mimetics boost the cytotoxic potential of a panoply of chemicals, including CDK9 inhibitors^25^.

Our aim in the present study is to seek the suitability of Dinaciclib in a series of STS *in vitro* models as cell death inductor, fully characterizing the cellular response to treatment. We have found that Dinaciclib is capable of inducing cell death as single agent. The cellular context, particularly the Bcl-2 family balance, at every model is decisive for the precise behavior after Dinaciclib incubation. Our data support that Bcl-x_L_ inhibition status is central for treatment tolerance. Moreover, Bcl-x_L_ specific inhibitors synergize with Dinaciclib to avoid such tolerance.

## Results

### Dinaciclib induces cell death in STSs independently of CDK1 and CDK9 expression levels

**Figure 1:**
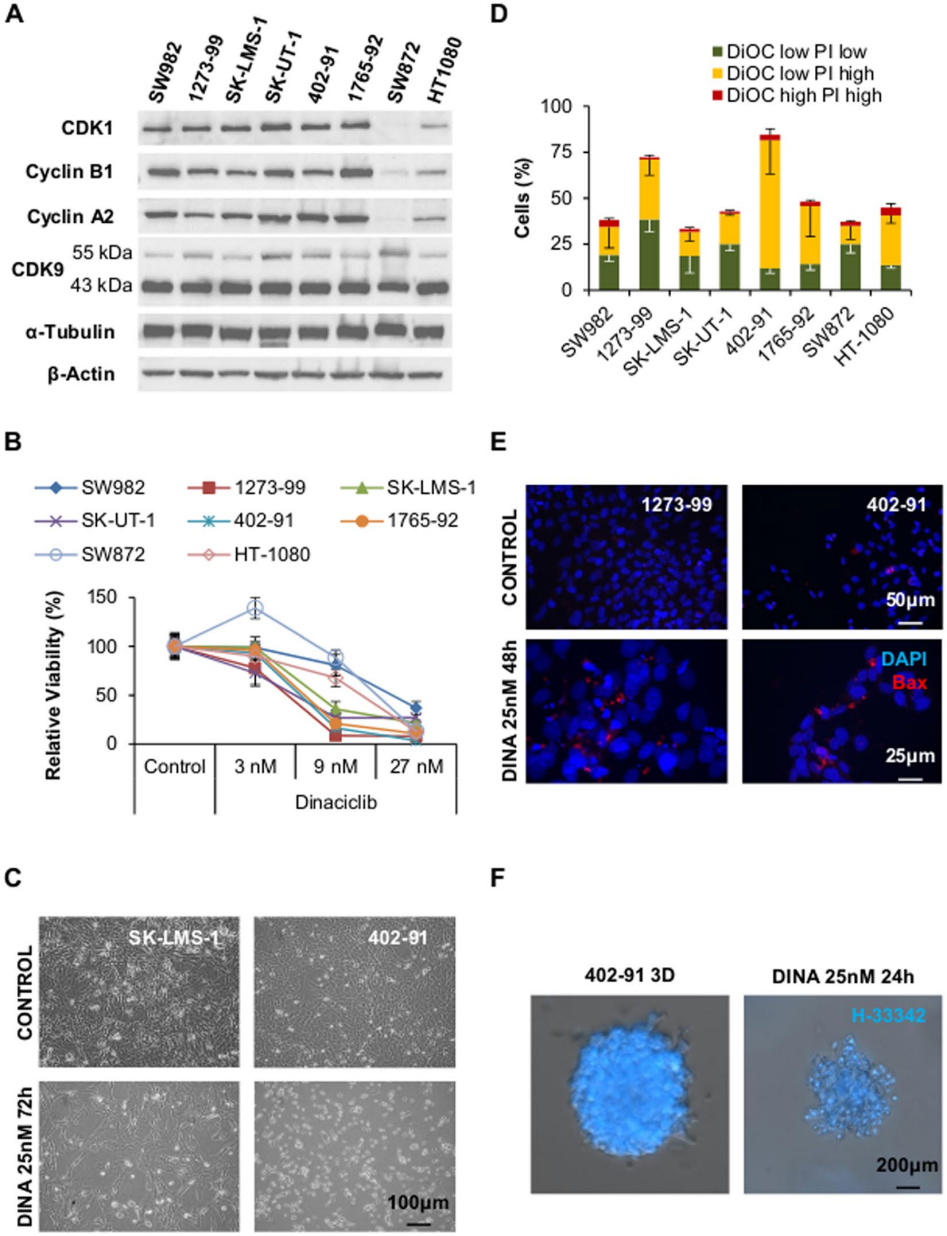
CDK inhibitor Dinaciclib is able to successfully inactivate sarcoma cell lines in a nanomolar range. (**A**) Representative western blot showing expression levels of Dinaciclib targets CDK1 and CDK9, and CDK1 partners Cyclin B1 and Cyclin A2 among different soft-tissue sarcoma derived cell lines. (**B**) Viability, measured by means of WST-1 reduction test, in a set of different STS cell lines incubated with increasing concentrations of Dinaciclib for 72 h; comparing with cells treated only with vehicle. (**C**) Microscopic imaging showing different cell behavior among cell lines when treated with Dinaciclib. (**D**) Comparison of apoptotic induction, cytofluorometric measurement of mitochondrial vital dye DiOC and death marker PI, in different STS derived cell lines treated with 25 nM Dinaciclib up to 72 h reveals different sensitivity among the cell lines. (**E**) Immunofluorescent detection of pro-apoptotic protein Bax showing its accumulation in *puncta* (mitochondria) in sensitive cells treated with 25 nM Dinaciclib. (**F**) 402-91 cells cultured as 3D spheroids by embedding into a Matrigel^®^ matrix and treated by Dinaciclib show loss of size and accumulation of pyknotic nuclei as revealed by staining with Hoechst-33342 (H-33342).

To have a base for inferring the response of STS to Dinaciclib, we analyzed the expression of CDK9, CDK1 and its partners Cyclin B1 and A2 in a series of cell lines representative of major groups of STS: synovial sarcoma SW982 and 1273–99, leiomyosarcoma SK-LMS-1 and SK-UT-1, myxoid liposarcoma 402–91 and 1765–92, dedifferentiated liposarcoma SW872 and myxofibrosarcoma HT-1080 (Fig. 1A). Protein expression was variable among the cell lines, from the rather homogenous 43 kDa (lower band) of CDK9 to the more variable CDK1. SW872 expressed comparatively undetectable levels of CDK1 and its partners. However, the levels of the 55 kDa (upper band) of CDK9 were among the highest.

The same set of cell lines was used to test the ability of Dinaciclib to impair cellular viability (read as WST-1 assay, Fig. 1B). SW982, SW872 and HT-1080 showed fewer decreases in viability at low concentrations, but at 27 nM differences among the cell lines were reduced. IC_50_ calculations failed for SW872 and HT-1080 due to high disparity of the data (Supplementary Fig. 1A), but actual values should be close to the 12.760 nM obtained for SW982.

However, when observed under the microscope, the response to Dinaciclib was more diverse (Fig. 1C). Rather than any form of cell death, most cell lines (like the SK-LMS-1 in the image) showed a stop in proliferation with almost the same number of cells as seeded. They mostly present a natural control morphology and only few detached cells can be observed. Only 402-91 and 1273-99 showed an extensive accumulation of detached, dead cells. To clarify this dual effect, we performed an analysis of apoptotic induction by cytofluorometric quantification of the levels of the vital stains DiOC_6_(3) and propidium iodide (DiOC-PI). Comparison among the 8 cell lines treated with 25 nM Dinaciclib for 72 h evidenced the two major phenotypes from the microscopical observations (Fig. 1D). Only 1273-99 and 402-91 presented values of apoptotic cell death exceeding the 50%. Dinaciclib concentration of 25 nM was chosen under experimental conditions as the minimal value above which cell death reached a plateau as soon as 24 h of treatment either in responsive or in tolerant cell lines (Supplementary Fig. 1B).

In order to confirm that Dinaciclib induced mitochondrial apoptosis, we studied the translocation of pro-apoptotic protein Bax to the mitochondria (Fig. 1E) and the arising of 85 kDa band of PARP, a common caspase-3 activation reporter (Supplementary Fig. 1C). Both features were confirmed in the sensitive cell lines 1273-99 and 402-91. Furthermore, the addition of 40 µM pan-caspase inhibitor z-VAD-fmk to the system for 36 h was sufficient to abrogate the induction of cell death in both cell lines (Supplementary Fig. 1D,E).

Dinaciclib potential as cell death inducer was conserved also in 3D tissue simulation. 402-91 cells embedded in a Matrigel^®^ matrix grew as microspheres. By treating the microspheres with Dinaciclib for 24 h prior staining with vital dye H-33342, we clearly observed a reduction of the size of the spheres but also the accumulation of bright pyknotic nuclei generated by apoptosis (Fig. 1F).

Taken together, our results show the potential of Dinaciclib as inducer of apoptotic cell death in STS at nanomolar concentrations.

### Dinaciclib effects on cells last after wash-out

**Figure 2:**
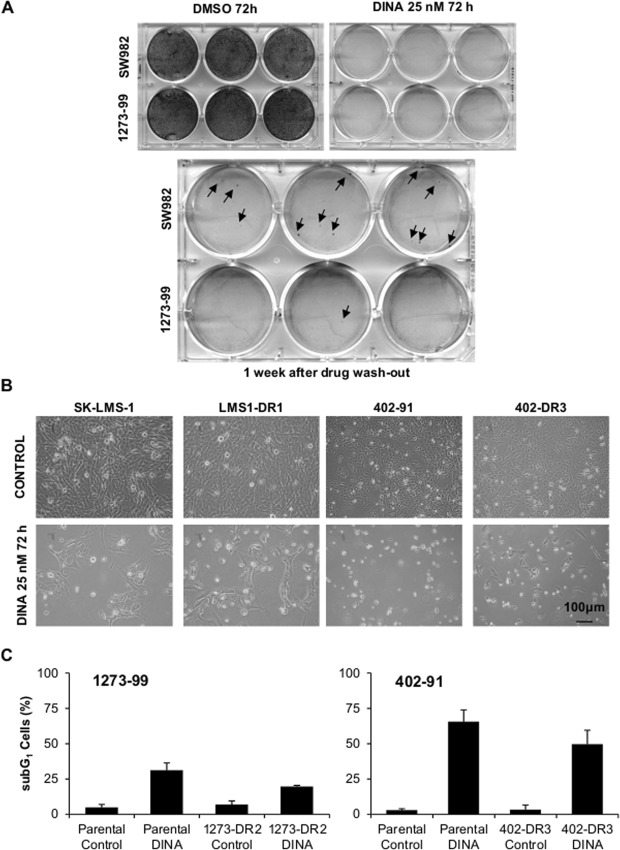
Dinaciclib did not drive strong selection of resistant clones after drug wash-out. (**A**) Crystal violet staining images showing resistant colonies (arrows) arising from highly sensitive 1273-99 cell line (low row) and more tolerant SW982 (upper row) after Dinaciclib wash-out. (**B**) Microscopic imaging showing polyclonal Dinaciclib Resistant (DR) strains response after being newly subjected to 72 h of treatment. (**C**) Cytofluorometric measurement of death induction (subG_1_ DNA content in PI profiles) for parental and derived DR strains from sensitive 1273-99 and 402-91 cell lines after a new cycle of 72 h incubation with Dinaciclib 25 nM (DINA).

To complete our understanding of cellular response to Dinaciclib, we performed long-term experiments analyzing cell response after drug wash-out. During the first days after Dinaciclib removal, cell lines from the tolerant group (like SW982) underwent cell death albeit the identification of a number of viable surviving colonies (Fig. 2A). Cells from the sensitive lines (like 1273-99) presented rare, if any, surviving colonies. Additionally, we allowed those colonies to grow into sub-cultures that we dubbed Dinaciclib Resistant (DR). Those DR lines were newly challenged with 72 h incubation of Dinaciclib 25 nM. DRs from the tolerant group did not show any evident difference in behavior from the parental culture. But those derived from the sensitive cell lines appeared to have acquired a better fitness against the drug (Fig. 2B). Nevertheless, when different experiments were summed up there was no statistical significance in this trend to a reduced cell death induction (Fig. 2C).

Therefore, our data suggests that Dinaciclib exerts a long-term effect in cell viability with the added value that surviving colonies do not show a strong selection towards drug resistance.

### Sensitive and tolerant cell lines differ in cell death protein expression patterns

**Figure 3:**
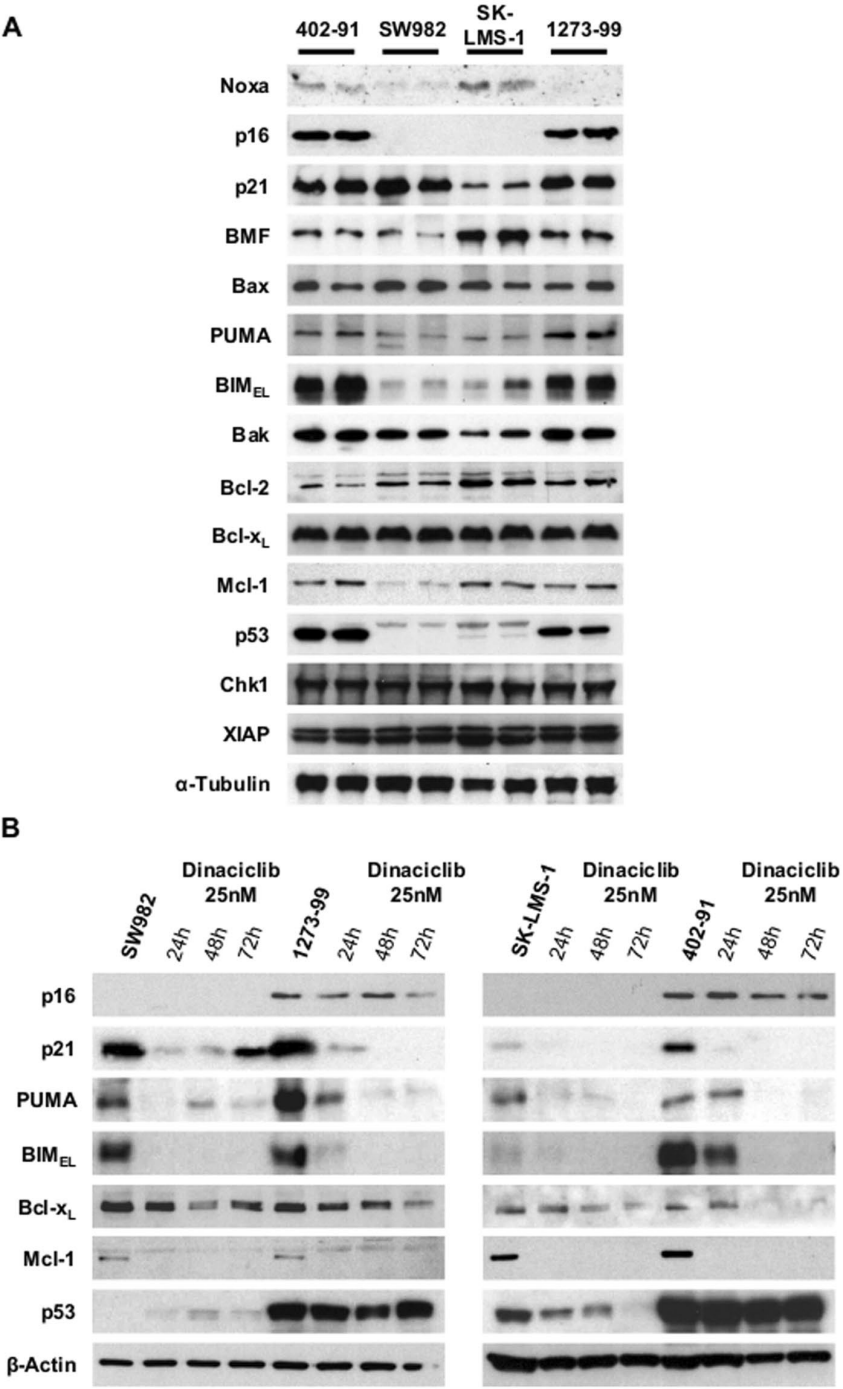
Dinaciclib highly sensitive cell lines present characteristic levels of key regulators of the cell cycle and apoptosis triggering. (**A**) Representative western blots showing protein expression patterns in control duplicates from myxoid liposarcoma 402-91, synovial sarcoma 1273-99 and SW982 and leiomyosarcoma SK-LMS-1. A representative sample of equal charges marker α-tubulin is also showed. (**B**) Representative western blots showing the decay rhythm of key components of cell cycle and apoptosis regulation at selected end-points of Dinaciclib incubation. A representative sample of equal charges marker β-actin is also showed.

As the known targets of Dinaciclib yielded no clear correlation with drug response, we performed an extensive analysis of proteins involved in cell cycle control among examples of responsive (402-91 and 1273-99) and tolerant (SW982 and SK-LMS-1) cell lines (see Fig. 1D). For most proteins (Noxa, BMF, Bax, Bak, Bcl-2, Bcl-x_L_, Mcl-1, Chk1 or XIAP) no significant associations could be made among their expression levels and the tolerance to Dinaciclib (Fig. 3A). However, other proteins did show remarkable differences in expression between the two groups.

Tumor suppressor p53 was almost undetectable in Dinaciclib tolerant cells when compared with the responsive ones (Fig. 3A). SK-LMS-1 expressed also relatively low levels of p21^Waf1/Cip1^ (p21) protein, a p53 target. In this regard, it is important to note that SK-LMS-1 cell line has been described as *TP53* mutant^26^. If we compare this data with the study of cell cycle changes, we can see that only cell line SW982 presented a statistically significant arrest in G_2_/M phase (Supplementary Fig. 2A). Although most of the Dinaciclib tolerant cell lines did share the same trend. To analyze the precise nature of this G_2_/M blockade we visualized treated and untreated cells immunolabelled for Ser10-phosphorylated Histone H3 (p-H3) and p21. Our results confirmed that the arrest was not due to accumulation of mitotic events but rather to the arrest of cells in the G_2_ phase (or in a tetraploid G_1_), as no mitotic figures were observed in 72 h treatment samples (Supplementary Fig. 2B). Besides, in concordance with the protein expression data (Fig. 3A), p21 was not induced in SK-LMS-1 cells (Supplementary Figure 2B).

CDK4 inhibitor p16^INK4a^ (p16) presented an expression pattern very similar to p53, being almost absent in Dinaciclib-tolerant cell lines. To complete the analysis, we found that senescence-related p16 translocated towards the nuclei in tolerant cell lines treated with Dinaciclib, in a way similar to the sensitive ones (whose signal was clearly more intense) (Supplementary Fig. 2C). Thus, tolerance to Dinaciclib seems to be linked to the generation of a partial G_2_ arrest without a clear or sustained induction of senescence, as cells finally die in the following days (Fig. 2A).

Among the mitochondrial apoptotic control machinery, major differences in protein expression levels were found in the small pro-apoptotic BH3-only proteins PUMA and BIM (Fig. 3A). Both were found comparatively over-expressed in Dinaciclib sensitive cell lines (402-91 and 1273-99).

The major effect of Dinaciclib via inhibition of CDK9 is the interruption of mRNA transcription^16,27^. On this basis, we analyzed how our main targets evolved upon Dinaciclib incubation (Fig. 3B). As expected, most of the proteins showed a radical decrease in expression after Dinaciclib incubation. In concordance with the data from Supplementary Fig. 2B, p21 was clearly induced in SW982 cell line. More importantly, anti-apoptotic regulator Bcl-x_L_ expression seemed only comparatively slightly reduced by Dinaciclib in all the models but 402-91 cells, which showed clear Bcl-x_L_ repression after 48 h of treatment. In contrast, Mcl-1 was early and completely suppressed in all cell lines. Finally, Dinaciclib responsive cell lines retained for longer time the, already higher, levels of pro-apoptotic BIM and PUMA.

Summing up, differences in key proteins among STS cell lines can explain particular features of Dinaciclib response.

### Bcl-xL silencing enhances sensitivity to Dinaciclib in SW982 and SK-LMS-1 cell lines

**Figure 4:**
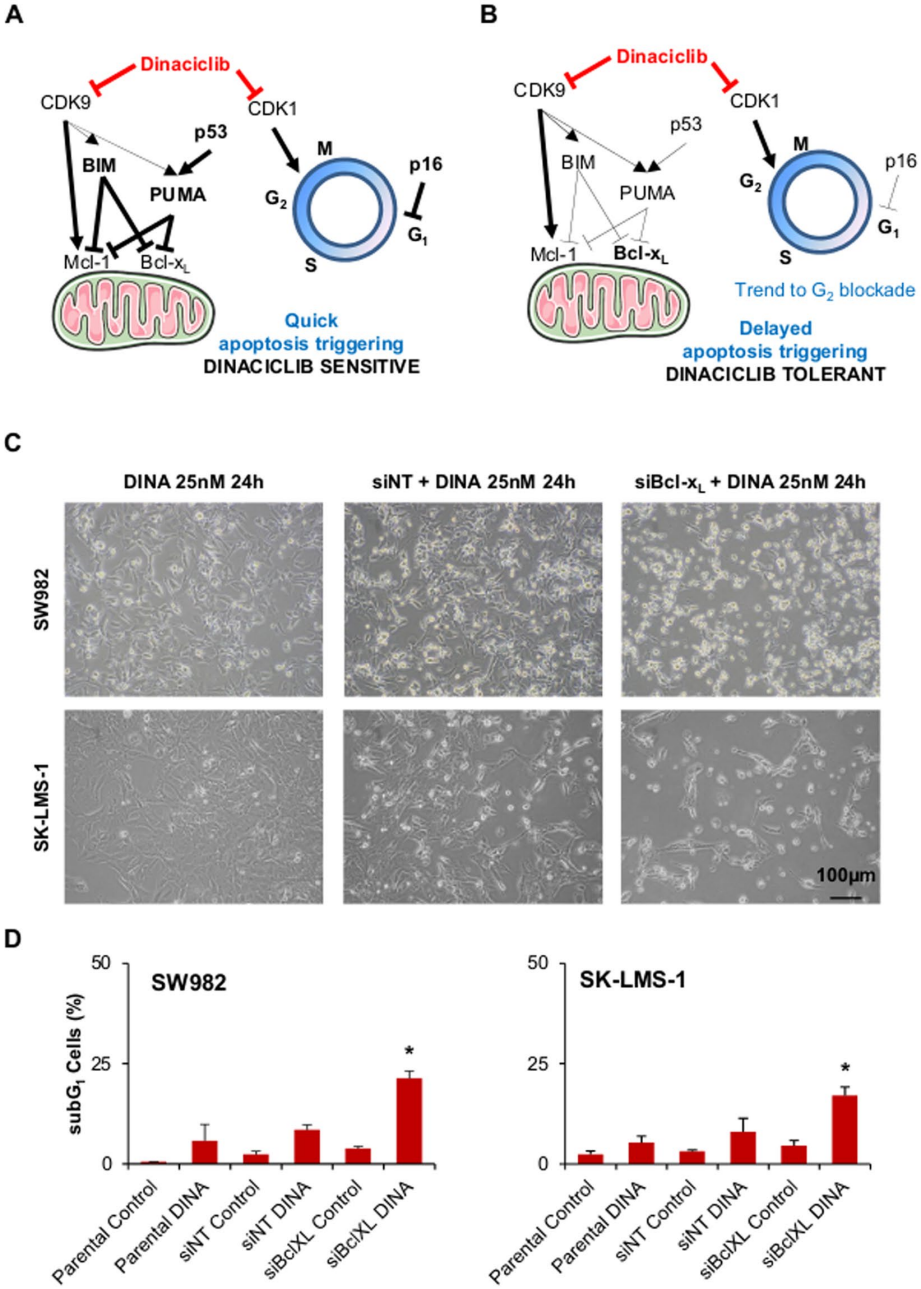
Bcl-x_L_ is the key regulator of apoptosis triggering after Dinaciclib treatment. (**A**,**B**) Schematics of the proposed mechanisms of action for the triggering of apoptosis in STS cell lines sensitive (**A**) and tolerant (**B**) to Dinaciclib. (**C**) Representative microscopic imaging of SW982 (above) and SK-LMS-1 (below) cell lines treated with 25 nM Dinaciclib for 24 h without prior challenge or after 24 h treatment with alternatively a non targeting (siNT) or a Bcl-x_L_ directed (siBcl-x_L_) siRNA. (**D**) Cytofluorometric quantification of cell death induction (measured as subG_1_ DNA content in PI profiles of fixed cell populations) after sequential treatment with siRNA and Dinaciclib. Data are presented as means ± SD. Statistical significance was achieved by the Student’s *t* test from at least three different experiments: *p ≤ 0.05.

Based on our protein expression data, we developed a hypothetical framework to study the mechanistic of Dinaciclib action. Dinaciclib sensitive cells undergo quick apoptosis due to their relative retention of higher levels of pro-apoptotic proteins (BIM, PUMA) after quick disappearing of anti-apoptotic Mcl-1 (visible 24 h after treatment, Fig. 3B). Their high levels of p16 (affecting the normal onset of G_1_ to S transition) prevent surviving cells to get arrested in G_2_ phase (Fig. 4A). On the other hand, Dinaciclib tolerant cells had relatively lower levels of BIM and PUMA. And they are lost faster after CDK9 inhibition than in the responsive ones (already undetectable after 24 h, Fig. 3B). Remaining Bcl-x_L_ is thus fully functional and prevents apoptosis to be triggered and cells proceed to G_2_ when other mechanisms (like p21 induction in SW982) are activated (Fig. 4B). In time, the induced damage will force the cells to die (Fig. 2A).

To check the coherence of our hypothesis, we have reviewed the data presented by Booher and co-workers in 2014 showing correlation between *MCL1:BCL2L1* mRNA ratio and viability after 24 h 100 nM Dinaciclib treatment^17^. By reducing the 250 samples to the ones appertaining to STS, we still found a statistically significant correlation, meaning that those cell lines more depending on Mcl-1 were also prone to die after Dinaciclib (Supplementary Fig. 3A).

For experimental validation, we addressed the role of Mcl-1 in Dinaciclib-induced cell death by siRNA silencing (Supplementary Fig. 3B). Although Mcl-1 was successfully depleted, no major increase in cell death was observed in SW982 cells (Supplementary Fig. 3C). However, depleting Bcl-x_L_ in Dinaciclib tolerant cell lines before Dinaciclib addition (Supplementary Fig. 3D) clearly resulted in an increase of the detachment of cells (Fig. 4C). When cell death induction was quantified, the increase of cell death at 24 h of treatment was deemed statistically significant (Fig. 4D).

Therefore, our results supported our hypothesis that Bcl-x_L_ is the more relevant counteracting mediator of Dinaciclib-induced apoptosis in STS.

### BH3-mimetics are able to synergistically enhance and accelerate cell death in STS

**Figure 5:**
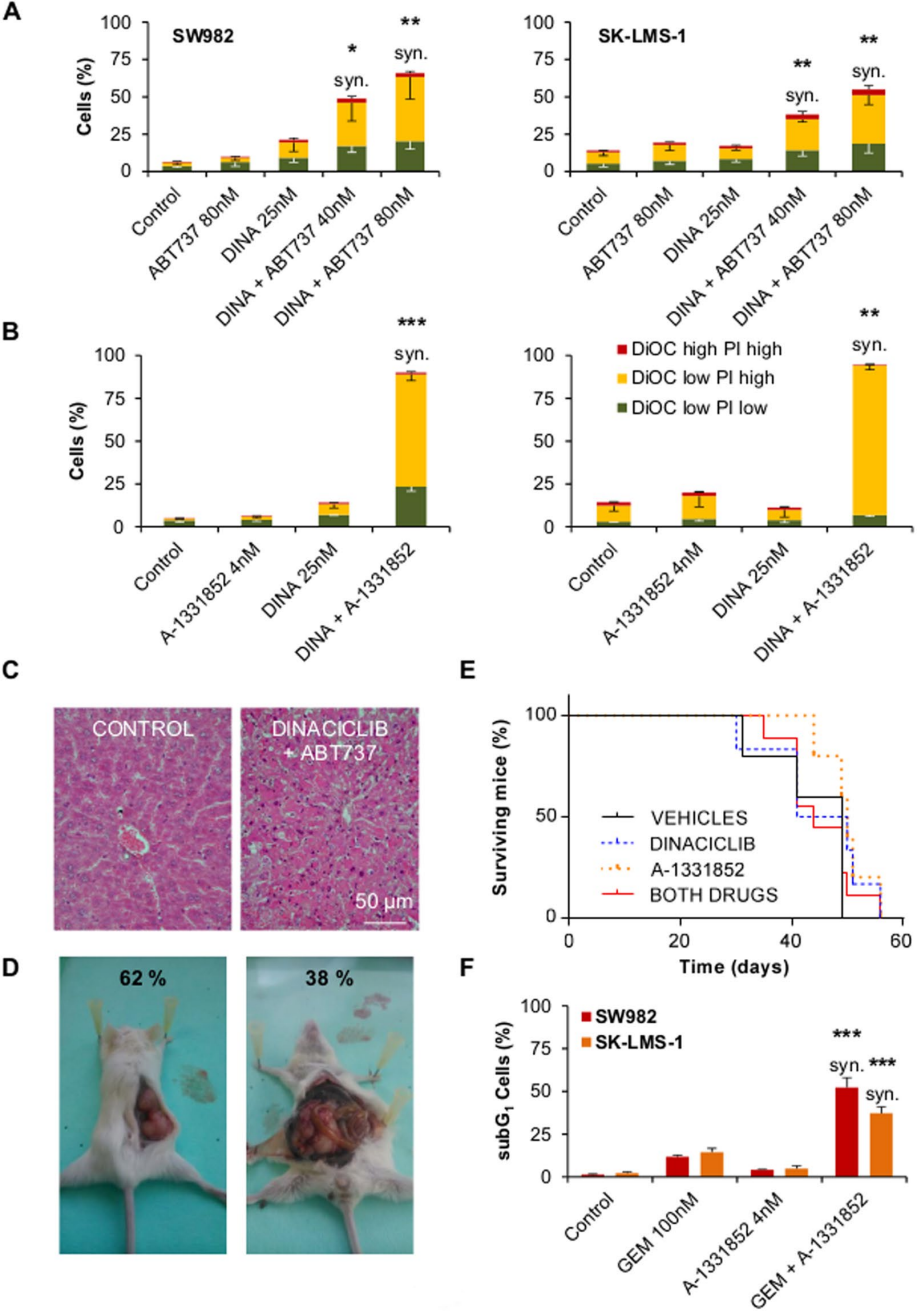
BH3-mimetics targeting Bcl-xL synergize with Dinaciclib to achieve quick apoptosis. (**A**) Cytofluorometric quantification of cell death via DiOC-PI staining of SW982 (left) and SK-LMS-1 (right) cell lines treated for 24 h with Dinaciclib 25 nM, ABT-737 80 nM or a combination of both drugs. (**B**) Cytofluorometric quantification of cell death via DiOC-PI staining of SW982 (left) and SK-LMS-1 (right) cell lines treated for 24 h with Dinaciclib 25 nM, A-1331852 4 nM or a combination of both drugs. (**C**) Microscopical imaging of H&E stained liver sections from Hsd:Athymic Nude*Foxn1*^*nu*^ mice control (left) and treated (right) after 3 i.p. injections with 40 mg/Kg Dinaciclib and 7 i.p. injections of ABT-737 who suffered sudden death at day 8. (**D**) Photographs showing typical subcutaneous tumor (left) and atypical intra-peritoneal tumor (right) caused by SK-LMS-1 cells in CB17.Cg-*Prkdc*^*scid*^*Lyst*^*bg-J*^/Crl mice. Percentages of occurrence are presented on the images. (**E**) Kaplan-Meier graph showing the survival of CB17.Cg-*Prkdc*^*scid*^*Lyst*^*bg-J*^/Crl mice inoculated (s.c.) with 5 × 10^6^ SK-LMS-1 cells and afterwards treated with i.p. 20 mg/Kg Dinaciclib, p.o. 25 mg/Kg A-1331852 or both drugs. Control mice were treated with both drugs vehicles. Time 0 represents the beginning of the treatment. (**F**) Cytofluorometric quantification of cell death (as subG_1_ in PI profiles in fixed cells) of SW982 and SK-LMS-1 cells treated with 100 nM Gemcitabine (GEM), 4 nM A-1331852 or a combination of both drugs for 48 h. Data are presented as means ± SD. Statistical significance was achieved by the Student’s *t* test from at least three different experiments: *p ≤ 0.05; **p ≤ 0.001; ***p ≤ 0.0001. syn. denotes synergistic behavior as determined according Valeriote & Lin.

To assess the possibility of a combination therapy to enhance Dinaciclib action we chose two different BH3-mimetics: one with a broad action spectrum, but excluding Mcl-1 (ABT-737) and one specific only for Bcl-x_L_ (A-1331852). BH3-mimetics activity over Dinaciclib tolerant cells as monotherapy was negligible (Supplementary Fig. 1A,C) but its effect was notable when combined with 25 nM Dinaciclib (Supplementary Fig. 4B,D). Cytofluorometric measurement showed that both combinations synergized^28^ in terms of apoptosis induction after 24 h of treatment at very low concentrations (Fig. 5A,B). Moreover, A-1331852 was more efficient than ABT-737. These results confirmed our operational hypothesis regarding Dinaciclib mechanism of action.

The next logical step was trying the drug combination *in vivo*. Previous to performing the *in vivo* studies, we checked *in vitro* the effects of the combination in immortalised myoblasts C2C12 as an appropriate model for the healthy tissue surrounding STSs. C2C12 cells treated with Dinaciclib + A-1331852 for 72 h showed no significant increase in cell mortality (Supplementary Fig. 5), indicating that the combination of drug had no deleterious effects on healthy cells, thus encouraging us to try the combination in *in vivo* experiments.

Our first approaches were aimed to assess treatment toxicity and cell growth using Hsd:Athymic Nude*Foxn1*^*nu*^ mice. We tried different combination regimes of Dinaciclib and ABT-737 based on literature^17,29^ (Supplementary Fig. 6A), unfortunately liver toxicity linked to massive hepatocyte death was observed in all the combinatorial cases (Fig. 5C). Moreover, human SK-LMS-1 grew poorly in athymic mice (Supplementary Fig. 6B). In consequence, we moved to a more immunodepressed CB17.Cg-*Prkdc*^*scid*^*Lyst*^*bg-J*^/Crl mice trying a lower dosage of Dinaciclib (due to its high toxicity) and A-1331852 instead of ABT-737 (Supplementary Fig. 6C). SK-LMS-1 cells grew better in these mice (Supplementary Fig. 6D), but an important percentage of tumors appeared as intra-peritoneal masses despite being injected sub-cutaneously (Fig. 5D). Intra-peritoneal tumors grew by surrounding, engulfing and finally invading the kidney (Supplementary Fig. 6E). Those mice were excluded from the final experiments. Final *in vivo* testing of anti-tumoral combined activity of both drugs was unsuccessful in terms of mice survival (Fig. 5E). The failure was attributed as a loss of action of the drugs (particularly Dinaciclib) due to the dosage reduction we have had to apply to avoid animal suffering. No change in body weight during the experiment was observed (Supplementary Fig. 6F). Summing up, we could not reach a therapeutic regime in which the drugs showed anti-tumor activity without induction of deadly toxicity.

Notwithstanding this set back, we sought to understand whether the key role of Bcl-x_L_ in Dinaciclib-induced cell death was a particular feature or part of a more general behavior. Other groups have already shown a positive cooperation of BH3-mimetics with CDK inhibitors and anthracyclines^25,30^. So, we tested another commonly used anti-tumor drug in STS therapy: nucleoside analogue Gemcitabine. Our results showed that A-1331852 synergized with Gemcitabine in terms of cell death induction after 48 h of combined treatment (Fig. 5F).

Taking together, these results unveil the specificity of cellular response to CDK inhibitor Dinaciclib and positively identify Bcl-x_L_ as the key player of apoptosis induction in these systems. More pharmaceutical research will be required to achieve a safe way for clinical practice, probably through the development of new delivery methods or new molecules with reduced side-effects. Still, this question falls beyond the scope of our study.

## Discussion

Dinaciclib is one of the most promising compounds in the group of CDK inhibitors because is able to act by meddling in the progression of late steps of the cell cycle, the entry in mitosis, transcriptional regulation and even the unfolded protein response^14,31,32^. It has been studied in clinical trials on cancer entities such as acute leukemia and other pediatric malignancies^11,13,19^. Dinaciclib was well tolerated, but its therapeutic effect as single agent did not achieve any total remission. However, in several neoplasias it is considered a good candidate for combination therapies^7,32,33^, including BH3-mimetics^25^.

Here we present new data on Dinaciclib pre-clinical results in STS cell lines. Our results show that Dinaciclib is able, as single agent, to impede cell proliferation in a nanomolar range (Fig. 1B) and to induce cell death at least in myxoid liposarcoma 402-91 and synovial sarcoma 1273-99 cells (Fig. 1D). Our model of 3D culture also proves that Dinaciclib is able to infiltrate and kill cells in solid tumors (Fig. 1F). These results align with previous pre-clinical reports that have also proved the utility of Dinaciclib in different tumor entities^15,17,32^. Moreover, our long-term studies after Dinaciclib removal showed that the deregulation induced by the drug was enough to promote a delayed death (five days after drug withdrawal) and that the remnant colonies did not develop an enhanced resistance to re-challenge (Fig. 2C). The lack of reinforced tolerance to Dinaciclib in DR lines opens the gate to the establishment of repeated administrations or combination procedures^34,35^.

To do so, a proper cell death mechanism characterization is paramount^36^. In this sense, our results proved that the main mechanism involved in Dinaciclib killing is the mitochondrial apoptotic pathway (Fig. 1D,E and Supplementary Fig. 1B–E). Other *in vitro* studies published with Dinaciclib also pointed to apoptotic mechanisms but they provided a less detailed characterization of the cell death^15,16,18^. Dinaciclib action involves two clearly different processes: cell cycle inhibition and alteration in protein production and homeostasis^9,31^. The study of those cell lines more tolerant to Dinaciclib is helpful as their delayed death allow us to observe other variables. Cell cycle profiling showed a trend to an arrest in G_2_-to-M transition that was only significant in SW982 (Supplementary Fig. 2A). SW982 cells are also the only cell line able to induce an increase in p21 protein (Fig. 3B and Supplementary Fig. 2B). Our data agreed to previous reports in other cancer entities, in which the cell cycle arrest was revealed when apoptosis was inhibited by Bax/Bak DKO^17^. But this mechanism may not be universal as most of our Dinaciclib tolerant cell lines did not reach statistically significant mitotic arrest. However, the sensitive cell lines indeed did increase their G_2_/M and postM (tetraploidy) phases when apoptosis was abrogated by z-VAD-fmk (data not shown). So, it appears that the effect on the cell cycle deregulation in our models may be an important contributor in the apoptotic triggering. Further research is needed to understand if other proteins (like p21) are required for G_2_ arrest after CDK1 inhibition.

The higher levels of tumor suppressor p53 in Dinaciclib sensitive cell lines (1273-99 and 402-91) is a key factor underlying Dinaciclib response, as it influences the levels of pro-apoptotic BH3-only proteins among other safety signalization processes^37^. On the other hand, the role of cell cycle regulator p16 is intriguing. It is one of the multiple transcripts of the *CDKN2A* locus, all of them with key functions in the homeostasis of the cell cycle, tumor suppression, senescence and aging^38–42^ The expression of p16 is not directly regulated by p53 but rather by Jun-B and Ras via Ets-1/2^38^. However, there are references cross-linking Ets-1/2 transcription factors with Bcl-2 family expression^43–46^ and even p53 activity^47^. In our settings, the sensitive cell lines 1273-99 and 402-91 exhibit higher levels of p16 than the tolerant ones (Fig. 3A and Supplementary Fig. 2C). Hence, there is supporting data to consider that expression levels of p16 could be linked to an apoptotic-permissive unbalance among the Bcl-2 family that enhances Dinaciclib sensitivity. As has been proved in other cancer entities, we consider interesting to explore the use of p16 as a predictive marker for cell cycle targeted therapies^39,42^.

Dinaciclib causes a general depletion of protein content in the cell via RNA Polymerase II dephosphorylation with different impact depending on protein distinctive half-life^7,9,16,17^. From our list of proteins of interest, we focused in Bcl-x_L_ as its levels decreased in a evident slow rate after Dinaciclib incubation (Fig. 3B). Delayed (48 to 72 h) depletion of Bcl-x_L_ was seen only in the most sensitive cell line (402-91). In contrast, PUMA and BIM levels decreased later in Dinaciclib responsive cell lines (48 h instead 24 h, Fig. 3B). Mitochondrial apoptosis is basically regulated by the inhibition status of large anti-apoptotic members of Bcl-2 family of proteins. This inhibition status relies on the balance with the pro-apoptotic members of the group, particularly the small BH3-only proteins^48^. Published literature has focused in anti-apoptotic regulator Mcl-1 levels as the main mediator of Dinaciclib cytotoxicity because its expression drops drastically after treatment^16–18^. Recently, a more comprehensive study from Inoue-Yamauchi and co-workers lead to a more complex panorama in which every member of the Bcl-2 family has a role in the apoptotic triggering^25^. For STS, the limited cohort from Booher and co-workers provides correlation between Dinaciclib sensitivity and Mcl-1/Bcl-x_L_ ratio (Supplementary Fig. 3A). However, our data showed that Mcl-1 levels are already low in SW982 cells (Fig. 3A) and quickly become almost undetectable upon Dinaciclib treatment in all cell lines (Fig. 3B). So, we envisaged a mechanistic hypothesis that put remnant Bcl-x_L_ interaction with BIM and/or PUMA in the decision point of apoptosis triggering in STS cell lines (Fig. 4A-B). When Dinaciclib is added, its effect over CDK9 causes a quick depletion in anti-apoptotic protein Mcl-1 but the effect over BIM or PUMA is weaker (and negligible on p53). PUMA and BIM are thus capable to initiate mitochondrial apoptosis by inhibition of Bcl-x_L_. Yet SW982 and SK-LMS-1 have low levels of BIM and PUMA. In the presence of Dinaciclib, the remaining levels of Bcl-x_L_ are enough to sustain cell viability for longer time. Then, the effects over the cell cycle (via CDK1) become detectable as a trend (significant in SW982) to a G_2_ blockade. Silencing experiments with siRNA confirmed our suspicion, as Bcl-x_L_ induced a clear increase in detached cells whereas Mcl-1 inhibition exerted no major difference (Fig. 4C,D and Supplementary Fig. 3B,C).

Bcl-x_L_ involvement as key mediator of apoptosis triggering has been previously described in several contexts including cell cycle alterations and other sarcoma entities^49–51^. Among the different Bcl-2 family interactions, Bcl-x_L_ has strong affinity for pro-apoptotic BH3-only proteins PUMA and BIM^49,50,52,53^. BH3-mimetics are a new class of drugs precisely designed to benefit of this interaction to promote cell death^23^. Since the recent FDA approval of Venetoclax, these molecules are in the spotlight of cancer research, albeit some groups are calling to improve the accurateness of data analysis^54^. A more detailed research in their precise mechanism of action is needed in order to design meaningful combinations for treatment. Nevertheless, we decided to go ahead and test our working hypothesis by designing drug combination testing *in vitro*. Two BH3-mimetics were chosen: ABT-737, a rather generic compound that, inhibits Bcl-2, Bcl-w and Bcl-x_L_ proteins^55^ and A-1331852, a newer and Bcl-x_L_ specific molecule^56^. Neither of them induced a relevant cell death accumulation as monotherapy, but when combined with Dinaciclib they showed a fast synergistic induction of cell death in both SW982 and SK-LMS-1 cells (Fig. 5A,B and Supplementary Fig. 4). The effects were clearly better with A-1331852 than with ABT-737, providing more support to our mechanistic hypothesis. Regretfully, we encountered too many problems at escalating our settings to *in vivo* experimentation (Fig. 5C–E and Supplementary Fig. 6). The main issue was the development of deadly liver toxicity, that was already glimpsed in literature^57^. Our data supports the need of more pharmaceutical research in order to generate a safer treatment protocol.

In the past few years, other groups have recognized the potential of BH3-mimetics in combination approaches to kill cancer cells. As previously commented, Inoue-Yamauchi and co-workers, using screening technologies in a small-cell lung cancer model, found that anti-tumor properties of CDK9 inhibitors and anthracyclines were boosted when combined with those molecules^25^. Research in STS has also pointed to the suitability of such combinations. Possible combinations for BH3-mimetics include anthracyclines, histone deacetylase inhibitors, tubulin poisons and etoposide^30,58–60^. Here, we add to the list Dinaciclib and Gemcitabine (Fig. 5F) and we point that the major focus should be put in Bcl-x_L_ inhibitors. Both, our data and the previous literature support the conclusion that Bcl-x_L_ targeted molecules behave better than generic BH3-mimetics. The existence of a single molecular route to explain this multiple activity seems unlikely. Bcl-x_L_ has been identified as a key player of cell death triggering after mitotic arrest^50^. This can explain the synergy reported with tubulin poisons or other compounds that induced strong mitotic arrest. But, it is not the case with Dinaciclib, as we obtained no relevant mitotic arrest linked to the cell death triggering. The G_2_ blockage reported for SW982 occurs at 72 h and combined cell death is verified at 24 h. Thus, in our setting it is more relevant the quick loss of protein players once Dinaciclib has disturbed the process of protein synthesis. The higher life time of Bcl-x_L_ positions it as the cornerstone of apoptotic induction. For Gemcitabine we cannot discard that the mechanism proceeds more similarly to the observed for mitotic disruptors. In this case, combined action takes more time to be verified (48 h *vs*. 24 h with Dinaciclib) showing that BH3-mimetics did not exert a pro-apoptotic activity by themselves. Indeed, their function is simply to push towards cell death *after* another injury has been detected in the cell.

Taking together, we have extensively showed that Dinaciclib (or an improved CDK9 inhibitor) is a suitable candidate for clinical trials in STS. It is capable to effectively kill STS cell lines in a relevant fashion, without inducing a strong selection towards drug resistance. Moreover, when combined with BH3-mimetics its action gets boosted in a remarkable way. Pharmacological research is, however, required in order to address the elevated toxicity linked to this drug.

## Methods

### Cell culture

Cell lines (Table 1) were cultured in RPMI-1640, except for C2C12 which were grown in DMEM. Media were supplemented with 10% (v/v) heat-inactivated FBS, 1% (v/v) Penicillin-Streptomycin and 1% (v/v) HEPES (Gibco). All cell lines were incubated in an AutoFlow NU-4750 Incubator (NuAire) at 37 °C in a humidified atmosphere of 5% CO_2_ in air. Otherwise specified, plasticware were purchased from Jet BioFil.

**Table 1:** List of cell lines employed in the study.

Cell line	Origin	Provider
SW982	Synovial Sarcoma	ATCC
1273-99	Synovial Sarcoma	Dr. Carmen de Torres, *Hospital Sant Joan de Deu* (Esplugues de Llobregat, Spain)
SK-LMS-1	Leiomyosarcoma	ATCC
SK-UT-1	Leiomyosarcoma	CLS
402-91	Myxoid Liposarcoma	Prof. Roberto Mantovani (*Università degli Studi di Milano*, Milan, Italy)
1765-92	Myxoid Liposarcoma	Prof. Roberto Mantovani
SW872	Dedifferentiated Liposarcoma	ATCC
HT-1080	Fibrosarcoma	ATCC
C2C12	Immortalised Mouse Myoblasts	ATCC

Authenticity of the cell lines was routinely confirmed by STR profiling analysis done at qGenomics SL (Esplugues de Llobregat, Barcelona, Spain).

Exponential growing cells were used for all experiments. Depending on cell size and proliferation rates, 80,000–180,000 cells were seeded in 12-well plates (or 1–2 million cells in P100 dishes) and allowed to grow for 24 h prior to treatment. Cells were routinely observed and photographed by means of an IX70 inverted epifluorescence microscope (Olympus).

For 3D cultures, we seeded 80,000 cells mixed in 1:1 dilution of medium and Matrigel^®^ (Corning Inc.) in 24-well plates already coated with a solidified layer of pure Matrigel^®^. Cells were kept in these conditions for 72 h to allow microsphere formation prior to treatment. Cell-permeable DNA-binding dye Hoechst-33342 (Molecular Probes) was added (10 μg/mL final concentration in medium) for nuclei visualization.

### Chemicals and treatments

Dinaciclib (CAS 779353-01-4) was purchased from Selleckchem and diluted in DMSO (Sigma-Aldrich) to a 100 μM concentration. Used dilutions were made in complete medium. Pan-caspase inhibitor N-benzyloxycarbonyl-Val-Ala-Asp(O-Me) fluoromethyl ketone (z-VAD-fmk, CAS 187389-52-2) was purchased from ApexBio and diluted in DMSO to form a 50 mM stock solution. Used dilutions (40 μM) were made in complete medium. Generic BH3-mimetic ABT-737 (CAS 852808-04-9) was obtained from ApexBio and diluted in DMSO to an initial 10 mM stock. The Bcl-x_L_ specific inhibitor A-1331852 (CAS 1430844-80-6) was purchased from ChemieTek and a 3 mM initial stock solution in DMSO was employed. The nucleoside analog Gemcitabine (CAS 122111-03-9) was obtained as hydrochloride salt from LC Laboratories and diluted in DMSO to form a 1 mM stock. In every case, further dilutions were made taking into account not to surpass 0.1% (v/v) content of DMSO in media.

### Proliferation and long-term clonogenic assay

For proliferation assessment, 3,000–5,000 cells were seeded in 96-well plates and treated with increasing concentrations of Dinaciclib, ABT737, A-1331852 or combinations of drugs for variable times up to 72 h. At desired time points, cells were incubated with WST-1 reagent (Roche) diluted 1:10 in complete medium up to 2 h in the incubator. Soluble formazan salt production from WST-1 was measured by absorbance (λ = 440 nm) in a PowerWaveXS plate reader spectrophotometer (BioTek Instruments). IC_50_ values were calculated using Prism software (GraphPad).

Long-term analyses of post-treatment survival were performed by seeding 180,000–300,000 cells in 6-well plates. Cells were cultivated with 25 nM Dinaciclib for 72 h prior drug wash-out and kept in culture up to a week. At that point cells were transferred to new plates to recover Dinaciclib Resistant strains or were stained with 0.5% (w/v) Crystal Violet (Sigma-Aldrich) solution in 1:5 Methanol:PBS for 20 min and washed with water. Images reflect representative results of at least three independent experiments.

### siRNA experiments

Cells were transfected using DharmaFECT (Dharmacom, GE HealthCare) following manufacturer’s instructions. ON-TARGETplus Non-Targeting Control Pool (Dharmacom) was used as reference and customized siRNA sequences were purchased from Sigma-Aldrich: 5′-AAUAACACCAGUACGGACGGG-3′ for Mcl-1 and 5′-ACAAGGAGAUGCAGGUAUUUU-3′ for Bcl-x_L_.

### Flow cytometry

For the simultaneous quantification of plasma membrane integrity and mitochondrial transmembrane potential (Δψ_m_), living cells were collected and stained with 1 μg/mL propidium iodide (PI, Molecular Probes) and 40 nM of DiOC_6_(3) (DiOC, Sigma-Aldrich) for 30 min at 37 °C. DiOC is a cyanine derivative that accumulates in functional mitochondria and dissipates when Δψ_m_ is lost. PI is a cell impermeable cationic stain (DNA intercalating) that accumulates in cell nuclei once the plasma membrane is no longer a barrier. So, we can identify cells committed to apoptosis (low levels of DiOC but still excluding PI), cells that suffered sudden necrosis (positive to PI but still retaining DiOC labeling) and cells that progress from apoptosis to secondary necrosis (negative to DiOC and positive to PI). Live cells (positive to DiOC and negative to PI) were not represented.

For cell cycle analysis, cells were fixed in 70% ice-cold ethanol and labeled with 50 µg/mL PI in PBS containing 500 µg/mL RNase (Sigma-Aldrich). This approach also allows cell death quantification as subG_1_ events. Cytofluorometric determinations were performed employing a Gallios flow cytometer and data were statistically evaluated using Kaluza software (Beckman Coulter). Only the events characterized by normal forward scatter (FSC) and side scatter (SSC) parameters were included in sub-sequent analyses.

### Western blot analysis

Cells were lysed with RIPA Buffer (ThermoFisher Scientific) containing Protease Inhibitor Cocktail Tablets and Phospatase Inhibitor Cocktail Tablets (Roche). Lysates were sonicated and centrifuged at 13,000 rpm for 20 min (at 4 °C). Protein content was determined with BCA assay (Pierce Biotech.). Lysate aliquots (50 μg) were resolved by 8–12% SDS-PAGE and transferred onto nitrocellulose membranes (Bio-Rad Laboratories). After blocking with 5% skimmed milk in PBS containing 0.2% (v/v) Tween-20 (Sigma-Aldrich) for 1 h (RT), membranes were incubated overnight at 4 °C with the appropriate primary antibody dilution (Table 2).

**Table 2:** List of primary antibodies employed for western blotting and/or immunofluorescence.

Target	Provider	Catalog number	Dilution
Noxa	Calbiochem	OP180	1:500
p16^INK4a^ (CDKN2A)	Abcam	ab108349	1:1000
Bax	Cell Signaling Technology	5023	1:2000
p21^Waf1/Cip1^	Cell Signaling Technology	2947	1:1000
PUMA	Cell Signaling Technology	12450	1:1000
Bmf	Abcam	ab181148	1:2000
Bak	Cell Signaling Technology	12105	1:2000
Bcl-2	Cell Signaling Technology	4223	1:1000
Bcl-x_L_	Cell Signaling Technology	2764	1:2000
CDK1 (cdc2)	Cell Signaling Technology	9116	1:1000
Mcl-1	Cell Signaling Technology	4572	1:1000
Cyclin A2	Cell Signaling Technology	4656	1:1000
Chk1	Cell Signaling Technology	2360	1:1000
XIAP	BD Transduction Laboratories	610762	1:5000
Cyclin B1	Cell Signaling Technology	4138	1:1000
BIM	Cell Signaling Technology	2933	1:1000
CDK9	Cell Signaling Technology	2316	1:1000
PARP	Cell Signaling Technology	9542	1:1000
phospho(Ser10)-Histone H3	Cell Signaling Technology	9706	1:1000

Blots were then incubated at room temperature (RT) for 1 h with Goat anti-Rabbit/anti-Mouse IgG (H + L) horseradish peroxidase–conjugated secondary antibodies (#31460 and #31430, Thermo-Fisher Scientific) and bands were revealed in Hyperfilm ECL (Amersham) by enhanced chemiluminescence employing ECL Western Blotting Substrate (Pierce Biotech.). Immunodetection of β-actin (#ab49900) or α-tubulin (#ab28439, both from Abcam) were used as loading reference.

### Immunofluorescence

Cells (30,000–80,000) were seeded over glass coverslips placed into 24-well plates prior to treatment. At desired end-points, cells were fixed by 30 min incubation in 4% (w/v) paraformaldehyde (Sigma-Aldrich) in PBS. Cells were sequentially permeabilized with 0.1% (w/v) SDS in PBS (USB Corp.) and subjected to antigen blocking with 10% (v/v) FBS in PBS (both for 20 min). Primary antibodies employed were Bax, p21^Waf1/Cip1^, p16^INK4a^ and phospho(Ser10)-Histone H3 (Table 2). Secondary antibodies were Goat anti-Mouse or anti-Rabbit IgG (H + L) Secondary Antibodies, conjugated either with Alexa Fluor^®^ 488 or 594 (ThermoFisher Scientific: #A-11001, #A-11005, #A-11008 and #A-11012). DNA counterstaining was performed upon 10 min incubation with 5 μg/mL solution of DAPI (Molecular Probes). Photographs were taken with either an Axio Observer.Z1 (Carl Zeiss AG) or a DM6000B (Leica Microsystems) epifluorescence microscope. Images were analyzed with Image J software (freely available from the National Institutes of Health at the address https://imagej.nih.gov/ij/).

### *In vivo* experimentation

For animal experimentation, Dinaciclib was purchased to ApexBio as powder and formulated in 20% hydroxypropyl beta-cyclodextrin (HPβCD, Sigma-Aldrich) in de-ionized water. The formulation was stored at 5 °C, used within 7 days and warmed to RT and vortexed for 3 sec before intraperitoneal (i.p.) administration. ABT737 was also obtained from ApexBio and formulated in 30% propylene glycol, 5% Tween 80, 10% DMSO, 3.3% dextrose (all from Sigma-Aldrich) in water. Aliquots were kept at RT and vortexed before i.p. administration. A-1331852 was provided by Abbvie under Material Transfer Agreement and formulated in 60% Phosal 50 PG (standardized phosphatidylcholine, PC, concentrate with at least 50% PC and propylene glycol; gently provided by AbbVie), 27.5% poly-ethylene glycol (PEG) 400, 10% ethanol, and 2.5% DMSO (all from Sigma-Aldrich). First, A-1331852 powder was suspended in DMSO and ethanol until a uniform cloudy suspension was obtained. PEG 400 and Phosal were then added and the solution was mixed by vortexing. The solution was allowed to sit for approximately 30 min after adding all the excipients helped to achieve a clear solution. A sonicator was also used for less than 10 min. Formulated compound was stored in an amber bottle at room temperature to protect it from light. A-1331852 formulation was administered *per os* (p.o.) by using an oral gavage. Dosages and administration regimes tested are summarized in Supplementary Fig. 6A–C.

Female Athymic (Hsd:Athymic Nude*Foxn1*^*nu*^) mice (n = 20) were purchased at Envigo. Female SCID^®^ Beige (CB17.Cg-*Prkdc*^*scid*^*Lyst*^*bg-J*^/Crl) mice (n = 40) were acquired at Charles River. Animal care procedures were followed according to the Institutional Guidelines for the Care and Use of Laboratory Animals. Ethics approval was provided by the institutionally appointed “IDIBELL Ethics Committee for Animal Experimentation”. IDIBELL animal facility abides to the Association for Assessment and Accreditation of Laboratory Animal Care (AAALAC) regulations.

Five million (5 × 10^6^) SK-LMS-1 cells in 1:1 RPMI:Matrigel^®^ were subcutaneously (s.c.) injected in the right dorsal flank of the mice. Tumor growth was followed by a caliper and volume was extrapolated using the formula vol = (L × l^2^)/2 were “L” refers to the major and “l” to the minor diameter measured. Drug treatments started when tumors reached a volume of approximately 100 mm^3^. Mice were blindly randomized into the different groups by using a 6-faced dice.

After the end of the experiment, tumors and organs of interest were extracted and fixed in 3.7% Formol solution in PBS (Sigma-Aldrich) for 24 h. Afterwards, samples were paraffin embedded and prepared for Hematoxylin-Eosin (H&E) staining following standard protocols. Microscopical imaging was performed by means of a Nikon Eclipse 80i workstation. Photographs were analyzed using GNU Image Manipulation Program (GIMP) software (freely available at http://www.gimp.org).

### Statistical Analysis

Unless otherwise stated, experiments were performed thrice. Data were analyzed for statistical significance using Student’s *t* test, using either Calc (The Document Foundation) or Excel (Microsoft Corporation) software; *p* ≤ 0.05 was regarded as significant. Synergistic behavior of drugs in combination was assessed by means of the comparison of the surviving fractions according to the method of Valeriote & Lin. Mice survival curves were analyzed using Prism (GraphPad Software Inc.).

## Supplementary information

Supplementary Information
