# Supplementary material for: Bcl-xL inhibition enhances Dinaciclib-induced cell death in soft-tissue sarcomas
Source: Sci Rep. 2019 Mar 7;9:3816. doi: 10.1038/s41598-019-40106-7 (PMC6405759; doi:10.1038/s41598-019-40106-7)

Bcl-x_L inhibition enhances Dinaciclib-induced cell death in soft-tissue sarcomas.

Santi Rello-Varona, Miriam Fuentes-Guirado, Roser López-Alemany, Aida Contreras-Pérez, Núria Mulet-Margalef, Silvia García-Monclús, Oscar M. Tirado and Xavier García del Muro

Suplementary Information

(A) Supplementary Figures:

Supplementary Figure 1 (related to Figure 1)**: Dinaciclib successfully induce apoptosis in sensitive cell lines.** (A) IC_{50} values obtained from WST-1 reduction test represented in Figure 1B. (B) Cytofluorometric measurement of mitochondrial vital dye DiOC and death marker PI showing apoptotic induction in 402-91 cell line (left), but not in SW982 cells (right), when treated with increasing concentrations of Dinaciclib for 24 h. (C) Representative western blots showing PARP protein cleavage after treatment with 25 nM Dinaciclib at different end times. (D) Representative microscopic imaging showing that Dinaciclib-induced apoptosis can be abrogated with co-incubation with 40 µM z-VAD-fmk for 36 h. (E) Cytofluorometric quantification of cell death (as $subG_1$ in PI profiles in fixed cells) of 1273-99 and 402-91 cells treated with 25 nM Dinaciclib and 40 µM z-VAD-fmk for 36 h. Data are presented as means ± SD. Statistical significance was achieved by the Student's *t* test from at least three different experiments: $^{**}p \leq 0.001$.

Supplementary Figure 2 (related to Figure 3)**: Differences in protein expression levels explain particular responses to Dinaciclib treatment.** (A) Cytofluorometric measurement of cell cycle phases by means of H-333258 profiling of DiOC high PI low (living) cells in control conditions or after 72 h treatment with 25 nM Dinaciclib (DINA). $SubG_1$ cells were excluded. (B) Microscopic imaging showing mitotic marker phosphorylated histone H3 (p-H3, arrowheads) and checkpoint protein $p21^{Waf1/Cip1}$ (p21, asterisks) in control conditions or after 72 h treatment with 25 nM DINA. (C) Microscopic imaging showing senescence marker $p16^{INK4a}$ (p16, asterisks) in control conditions or after 24 h treatment with 25 nM DINA. Data are presented as means ± SD.

Statistical significance was achieved by the Student's *t* test from at least three different experiments: *$p \leq 0.05$.

Supplementary Figure 3 (related to Figure 4)**: Mcl-1 is not involved in Dinaciclib tolerance in STS cell lines.** (A) Reanalysis of Boohrer *et al.* data on Dinaciclib tolerance and Mcl-1 and Bcl-x_L expression (mRNA) constrained to the "Soft-Tissue Sarcoma" subset. Cell lines names are noted. Rhabdomyosarcoma cell lines are underlined. Cell lines also included in our study are highlighted in bold. (B) Representative western blot showing Mcl-1 expression in SW982 cell line in the combined presence of siRNA constructs and Dinaciclib. (C) Representative microscopic imaging showing SW982 cell response to sequential silencing of Mcl-1 prior treatment with Dinaciclib 25 nM. (D) Representative western blot showing Bcl-x_L expression in SW982 and SK-LMS-1 in sequential silencing of the target prior of Dinaciclib incubation. Data are presented as means ± SD. Statistical significance was achieved by the Student's *t* test from at least three different experiments: **$p \leq 0.001$.

Supplementary Figure 4 (related to Figure 5)**: BH3-mimetics are unable to induce relevant harm to STS cell lines as monotherapy.** (A to D) Viability measured by means of WST-1 reduction test in SW982 (left) and SK-LMS-1 (right) cell lines after: (A) 72 h treatment with increasing concentrations of ABT-737; (B) 72 h treatment with increasing concentrations of ABT-737 combined with 25 nM Dinaciclib; (C) 24 h treatment with increasing concentrations of A-1331852; (D) 24 h treatment with increasing concentrations of A-1331852 combined with 25 nM Dinaciclib. Data are presented as means ± SD. Statistical significance was achieved by the Student's *t* test from at least three different experiments: *$p \leq 0.05$; **$p \leq 0.001$; ***$p \leq 0.0001$.

Supplementary Figure 5 (related to Figure 5)**: Combined treatment with Dinaciclib and BH3-mimetics shows limited toxicity *in vitro* in non-tumoral cells.** Cytofluorometric quantification of cell death (as subG_1 in PI profiles in fixed cells) of C2C12 immortalised myoblasts treated with 25 nM Dinaciclib and 4 nM A-1331852 for 72 h. Data are presented as means ± SD. Statistical significance was achieved by the Student's *t* test from at least three different experiments.

Supplementary Figure 6 (related to Figure 5)**: Combined treatment with Dinaciclib and BH3-mimetics shows deathly liver toxicity.** (A) Treatment regimes using Dinaciclib and ABT-737 in Hsd:Athymic Nude*Foxn1*^{nu} mice. Mortality rates are indicated. (B) Tumor success rate and growth curve for SK-LMS-1 cell line in Hsd:Athymic Nude*Foxn1*^{nu} mice. (C) Treatment regime with Dinaciclib and A-1331852 tested in CB17.Cg-*Prkdc*^{scid}*Lyst*$^{bg\text{-}J}$/Crl mice. (D) Tumor success rate and growth curve for SK-LMS-1 cell line in CB17.Cg-*Prkdc*^{scid}*Lyst*$^{bg\text{-}J}$/Crl mice. (E) Microscopical imaging of H&E staining of one intra-peritoneal sample tumor showing tissue transition from renal cortex to tumor mass. * indicate renal corpuscles. (F) Mean CB17.Cg-*Prkdc*^{scid}*Lyst*$^{bg\text{-}J}$/Crl mice weight variation during combination treatment with Dinaciclib and A-1331852. Time 0 represents the beginning of the treatment.

<u>(B) Original full blot images.</u>

From Figures:

1A

3A

3B

Supplementary 1C

Supplementary 3C

Supplementary 3D

A

	SW982	1273-99	SK-LMS-1	SK-UT-1	402-91	1765-99	SW872	HT-1080
IC_{50}	12.760	3.708	6.676	3.948	5.671	5.828	Ambiguous	Ambiguous
R^2	0.7975	0.9595	0.9736	0.8186	0.9552	0.9787	0.8199	0.8720

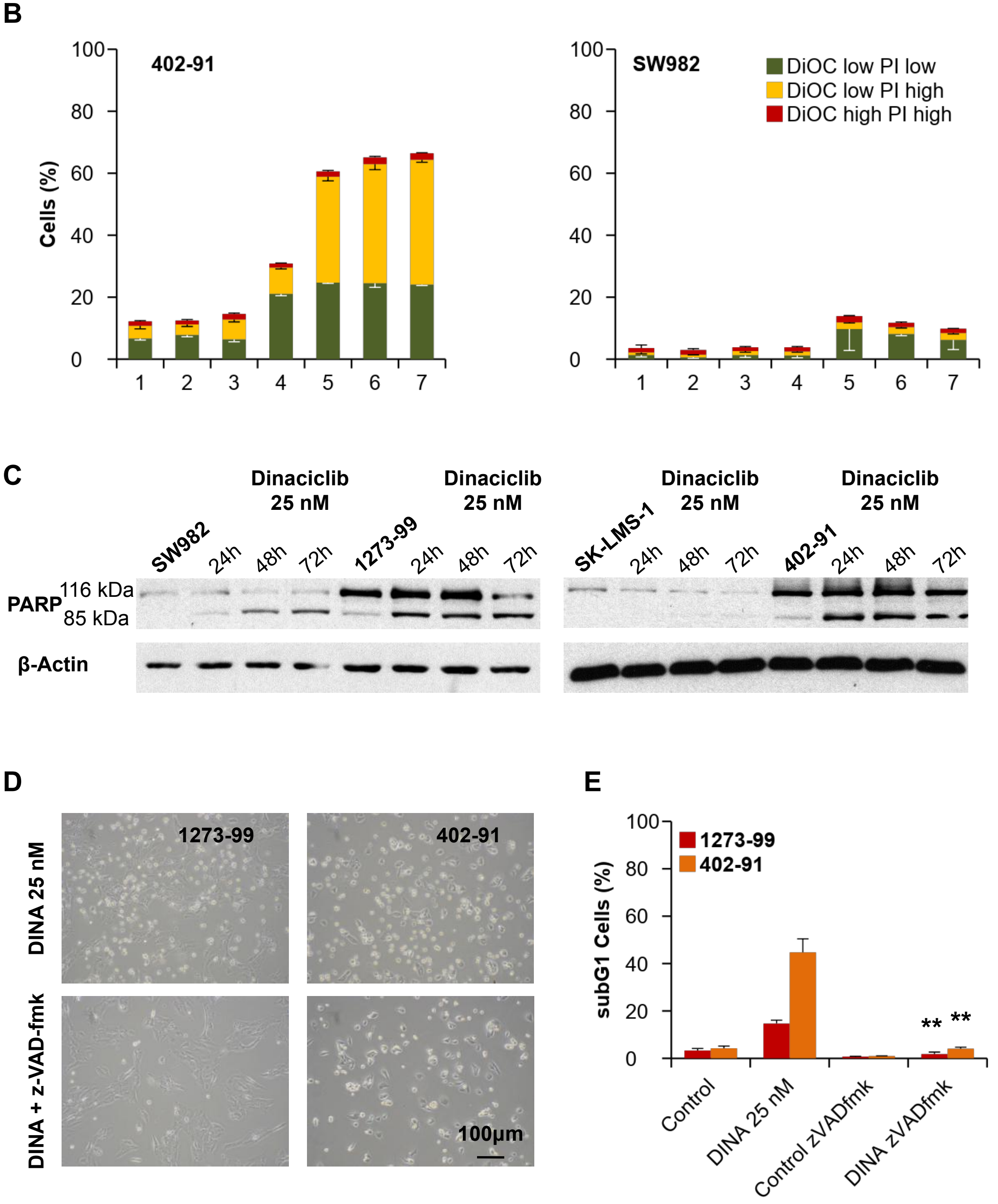

SuppFigure 1

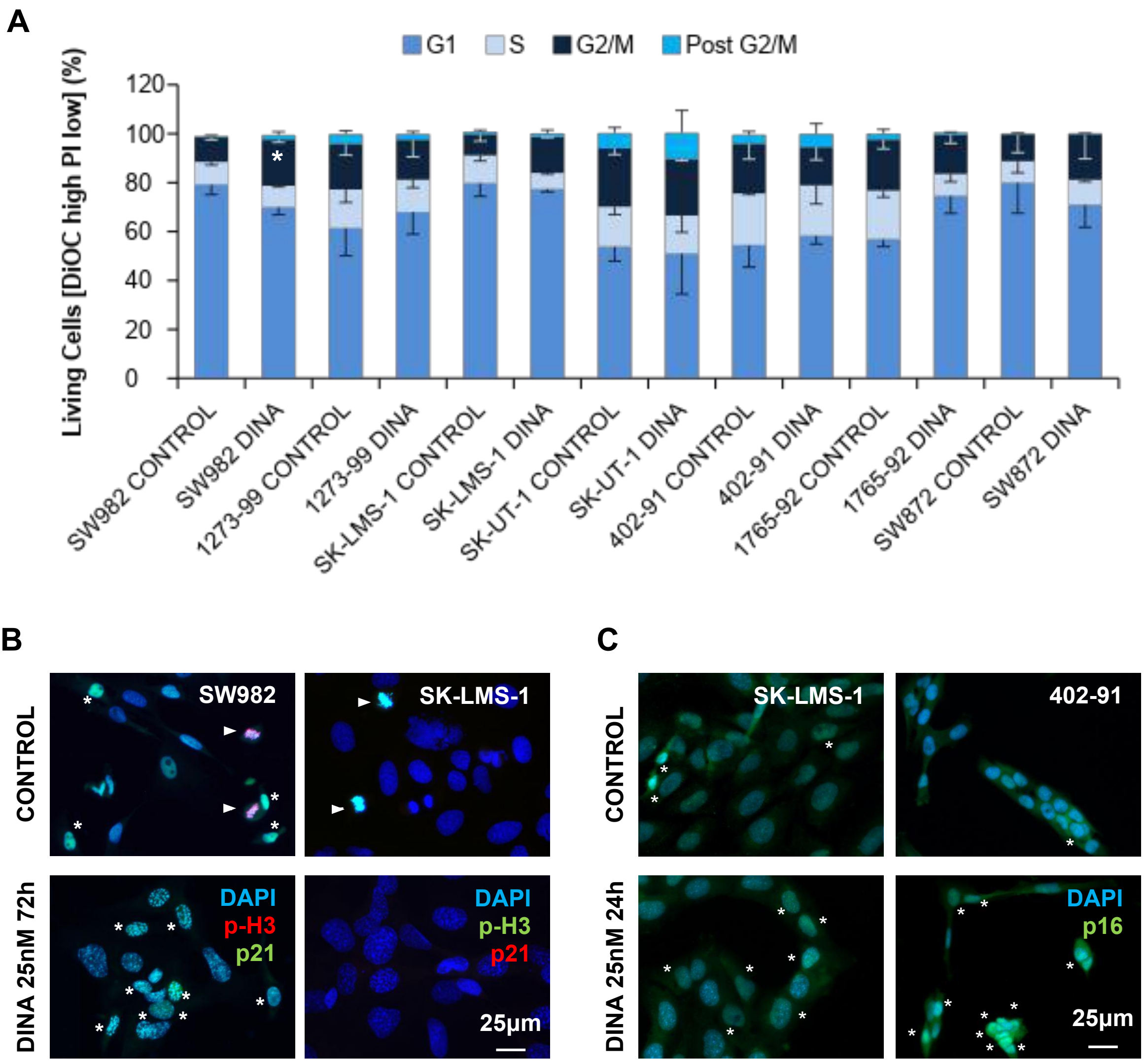

SuppFigure 2

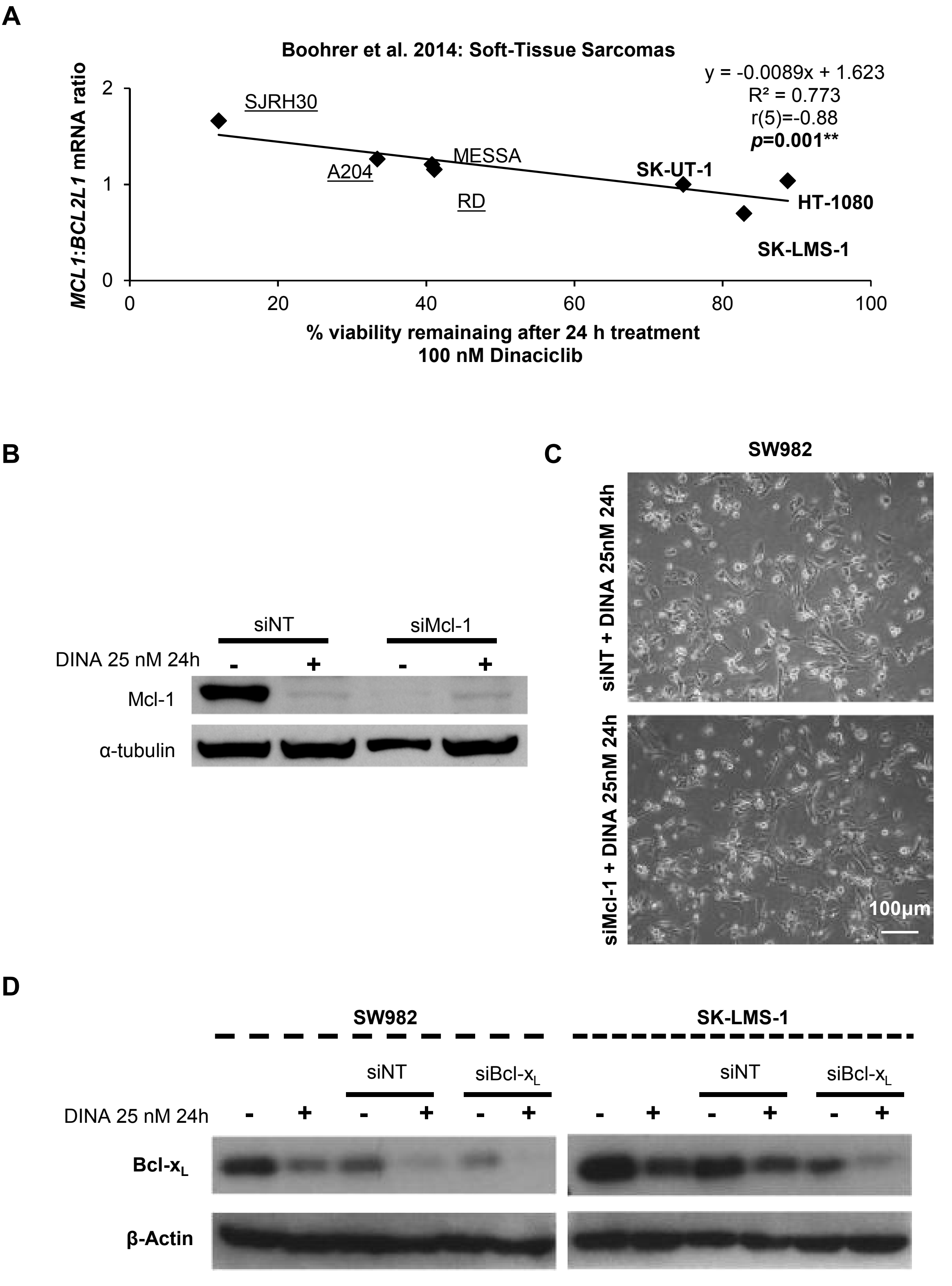

SuppFigure 3

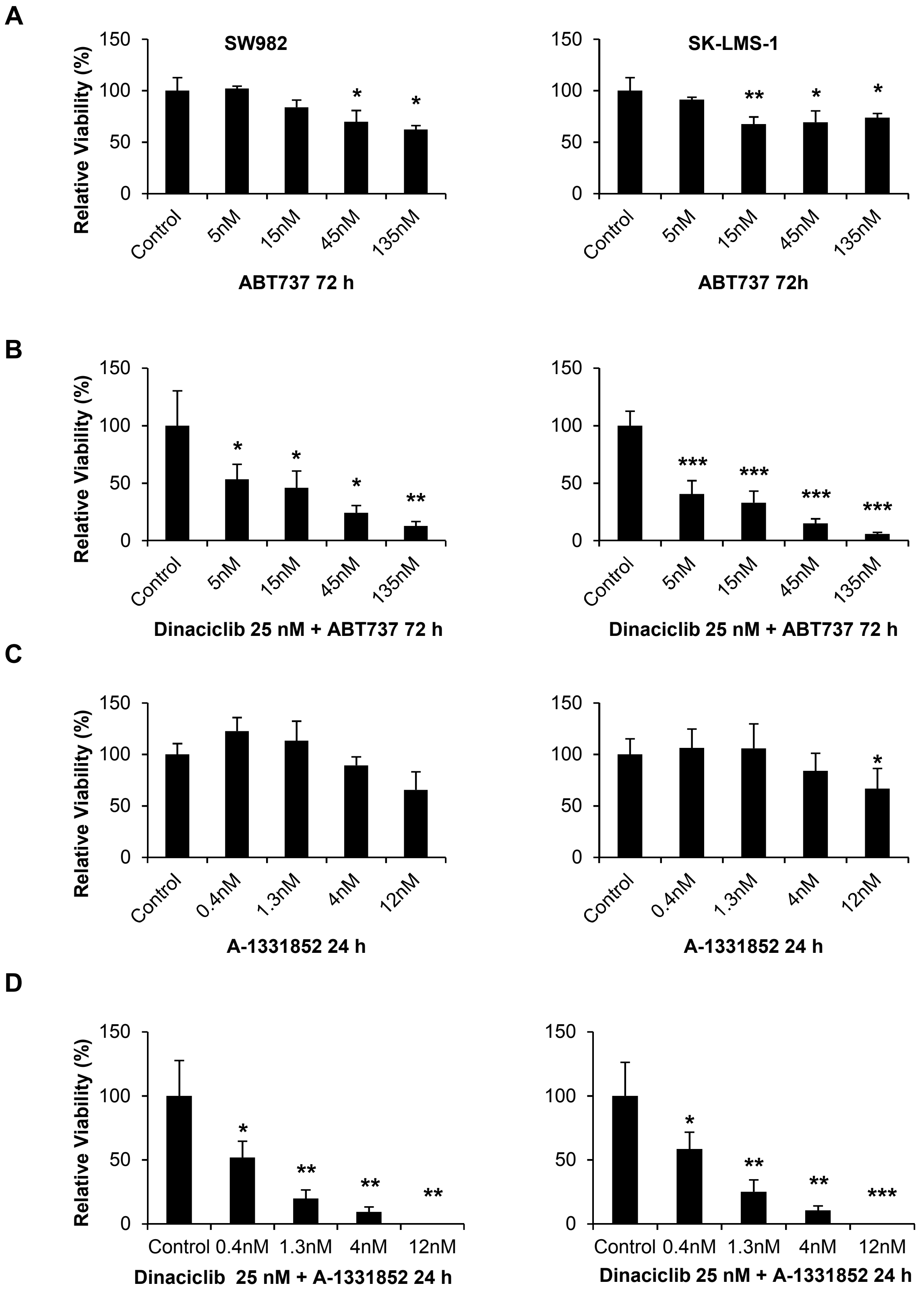

SuppFigure 4

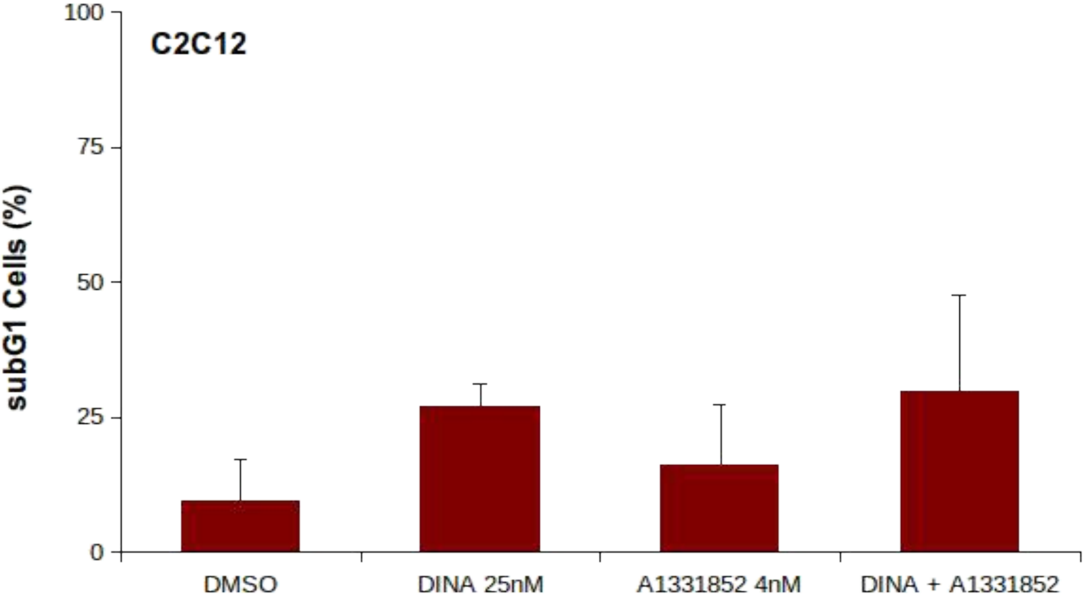

SuppFigure 5

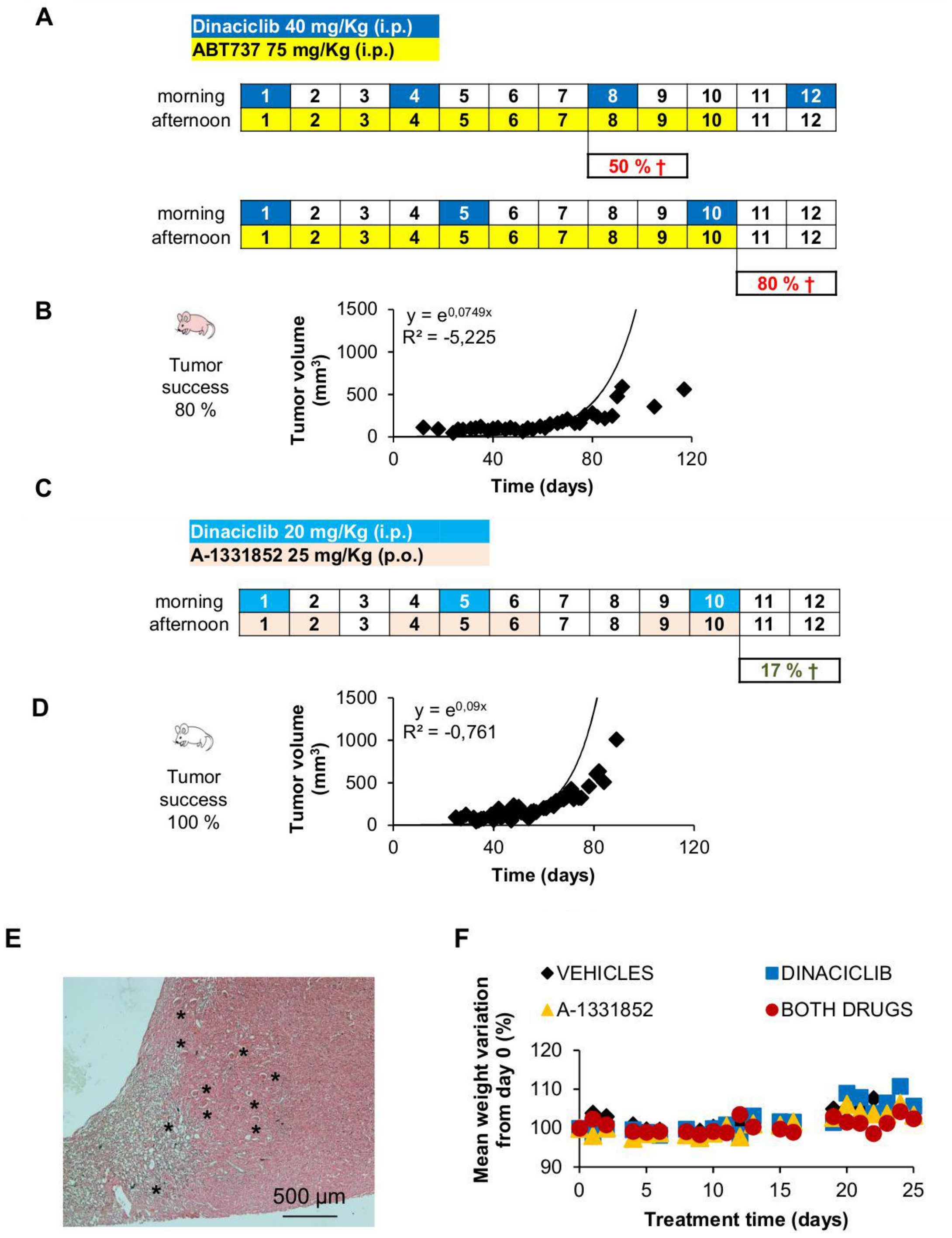

SuppFigure 6

Rello-Varona *et al.*:

Bcl-x_L interferes in Dinaciclib-induced apoptosis in sarcomas.

ORIGINAL FULL BLOT IMAGES

Figure 1A

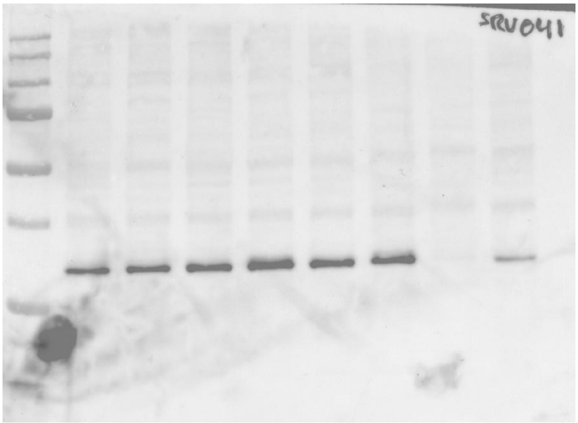

CDK1

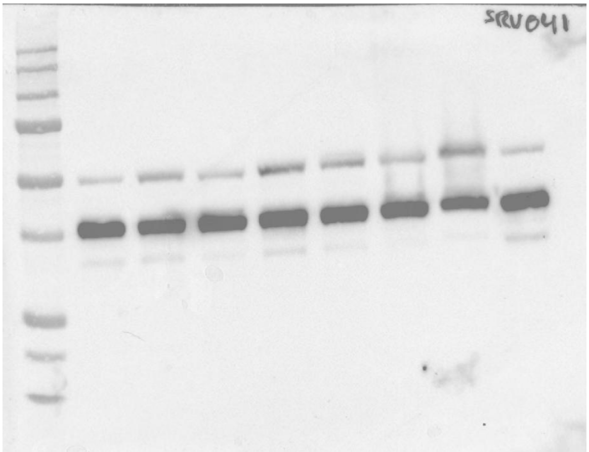

CDK9

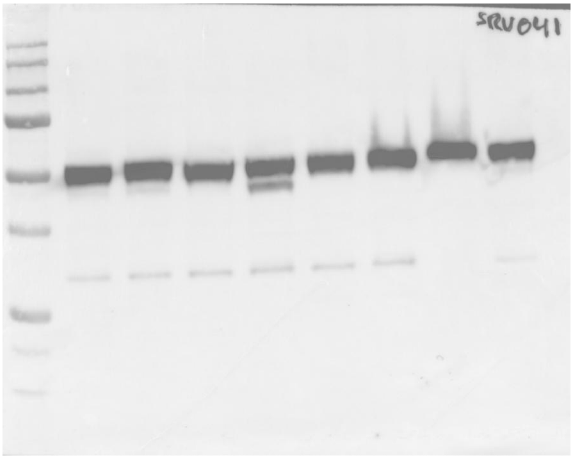

α-Tubulin

Rello-Varona *et al.*:

Bcl-x_L interferes in Dinaciclib-induced apoptosis in sarcomas.

Figure 1A

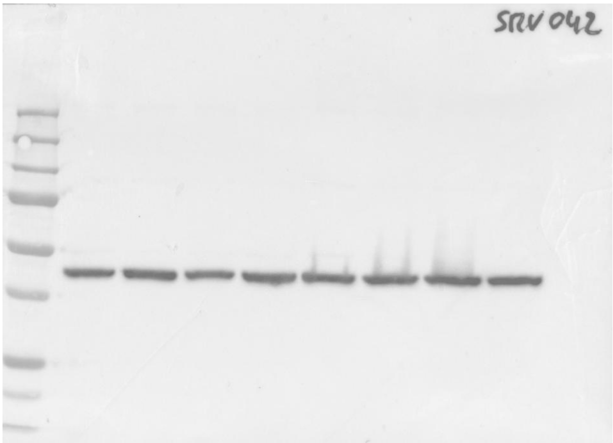

β-Actin

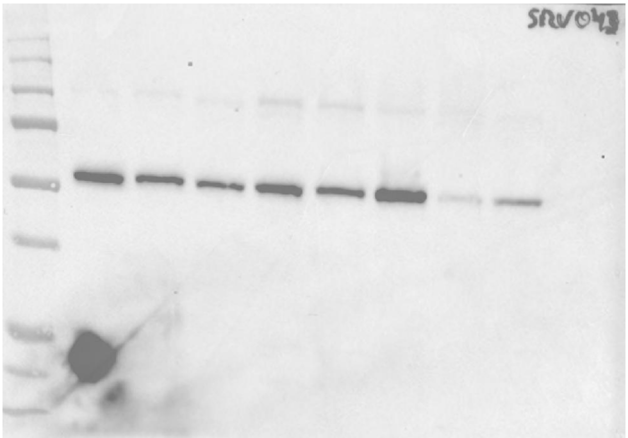

Cyclin B1

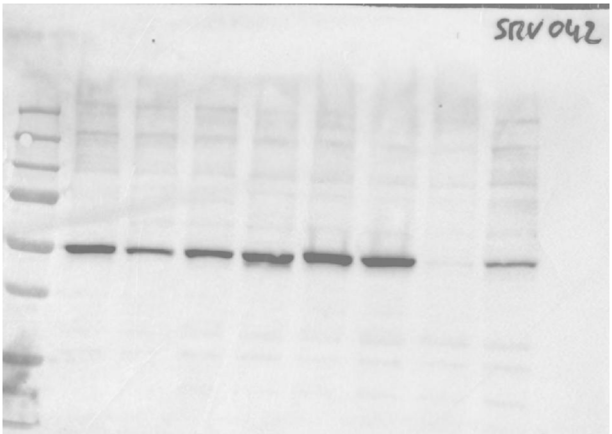

Cyclin A2

Rello-Varona *et al.*:
Bcl-x_L interferes in Dinaciclib-induced apoptosis in sarcomas.

Figure 3A

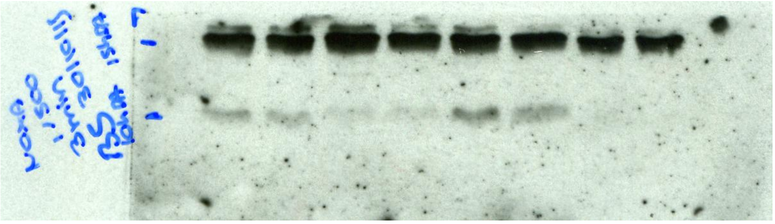

Noxa

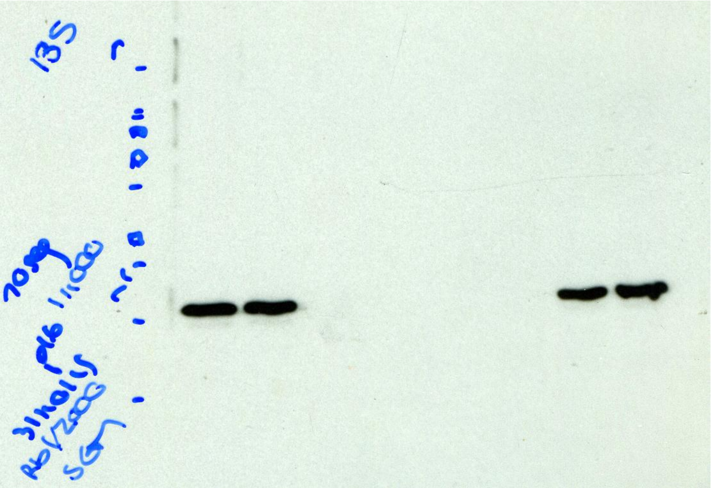

p16

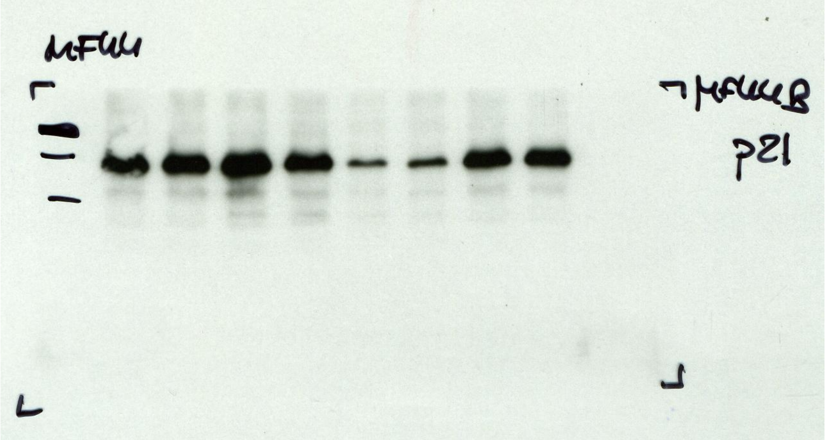

p21

Rello-Varona *et al.*:
Bcl-x_L interferes in Dinaciclib-induced apoptosis in sarcomas.

Figure 3A

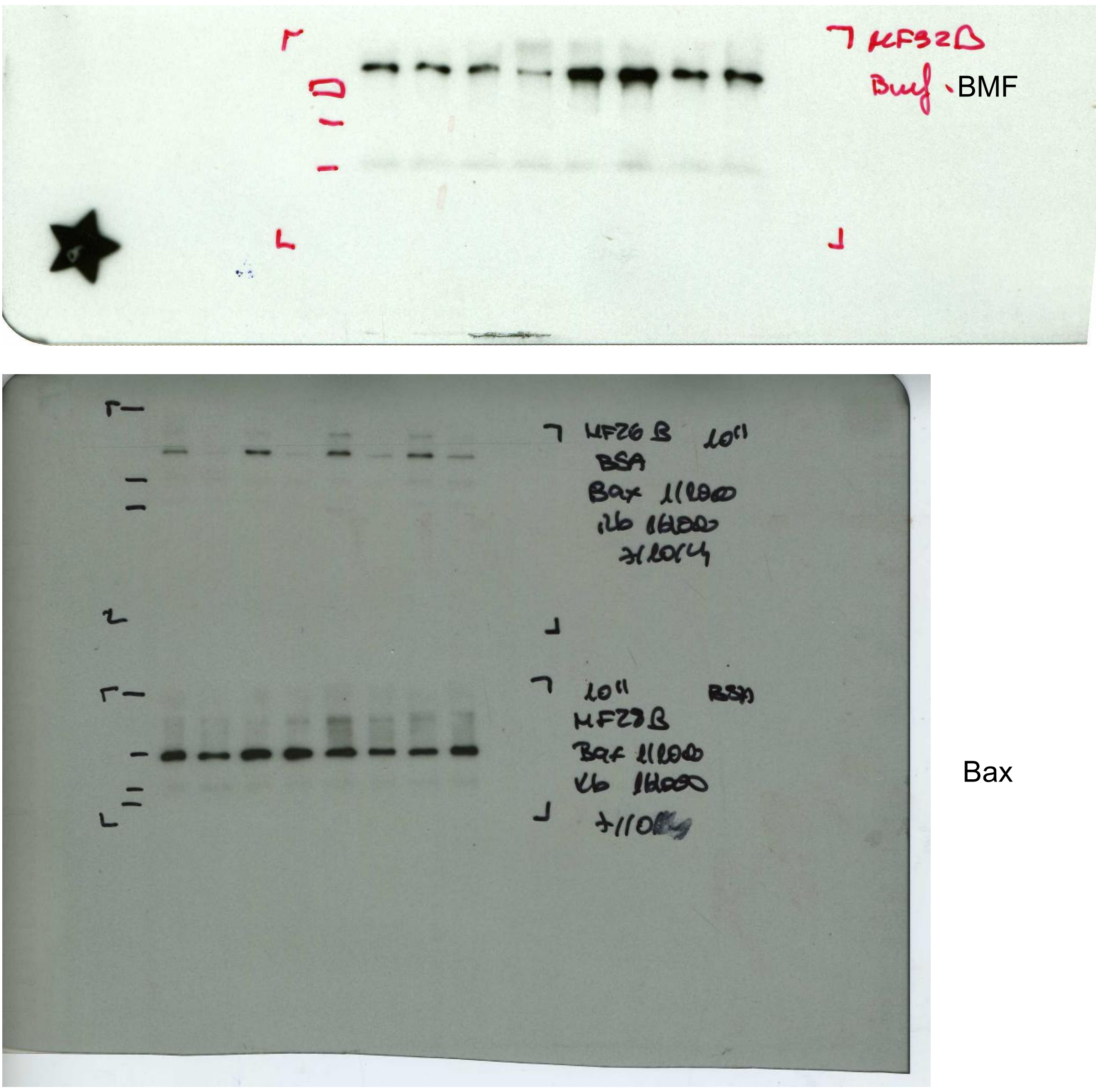

Rello-Varona *et al.*:

Bcl-x_L interferes in Dinaciclib-induced apoptosis in sarcomas.

Figure 3A

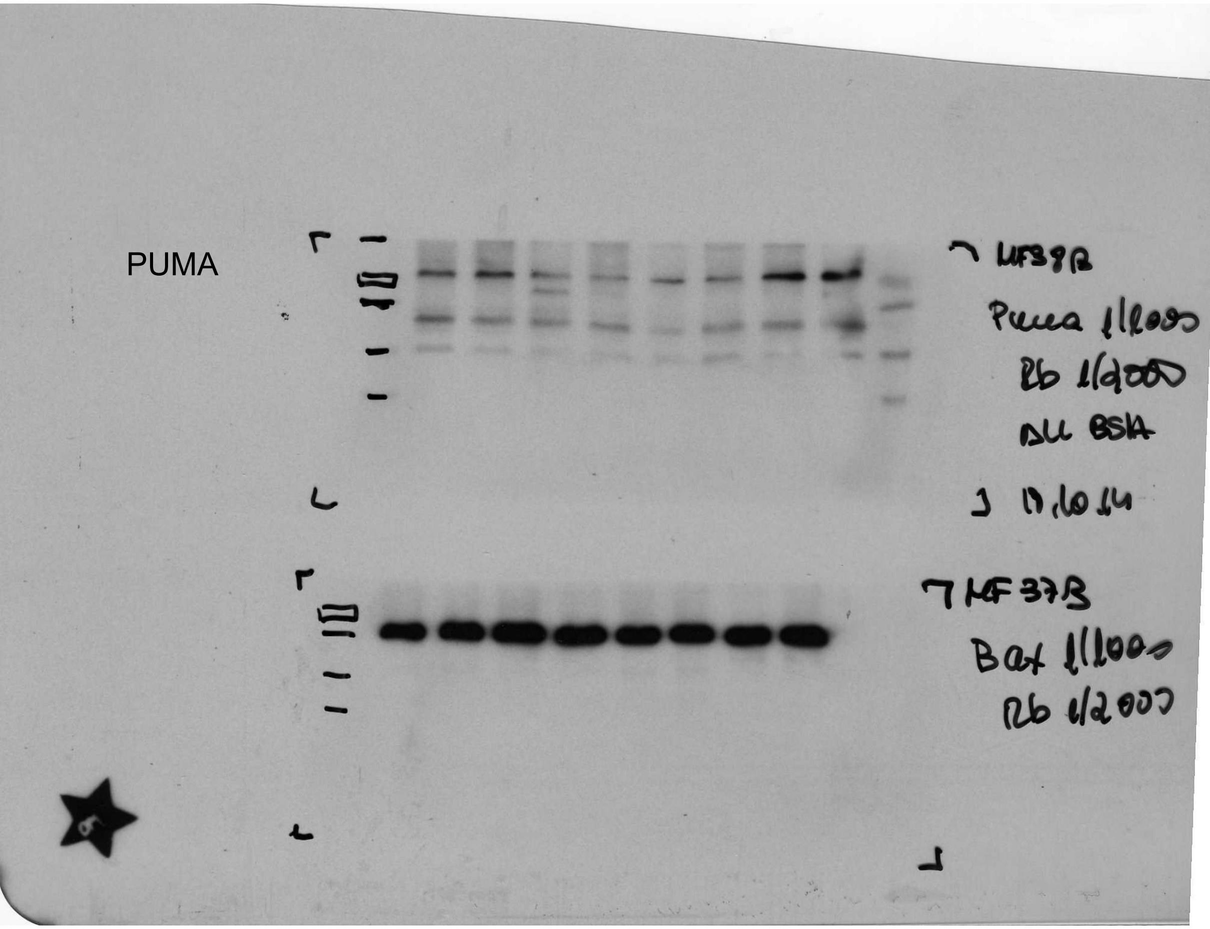

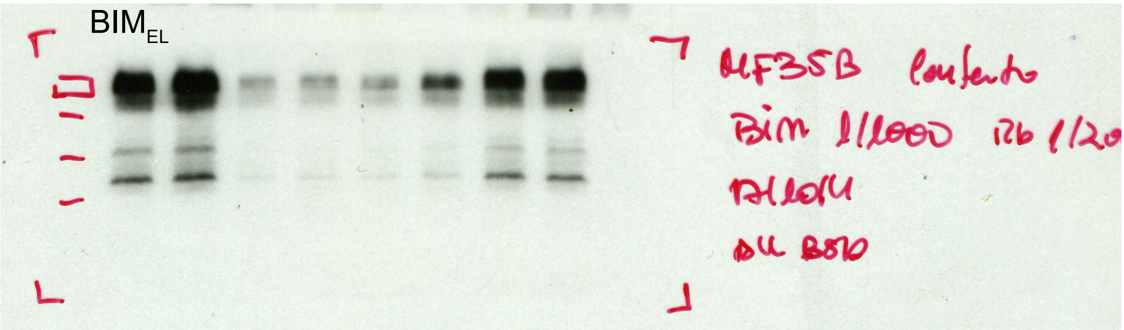

Rello-Varona *et al.*:

Bcl-x_L interferes in Dinaciclib-induced apoptosis in sarcomas.

Figure 3A

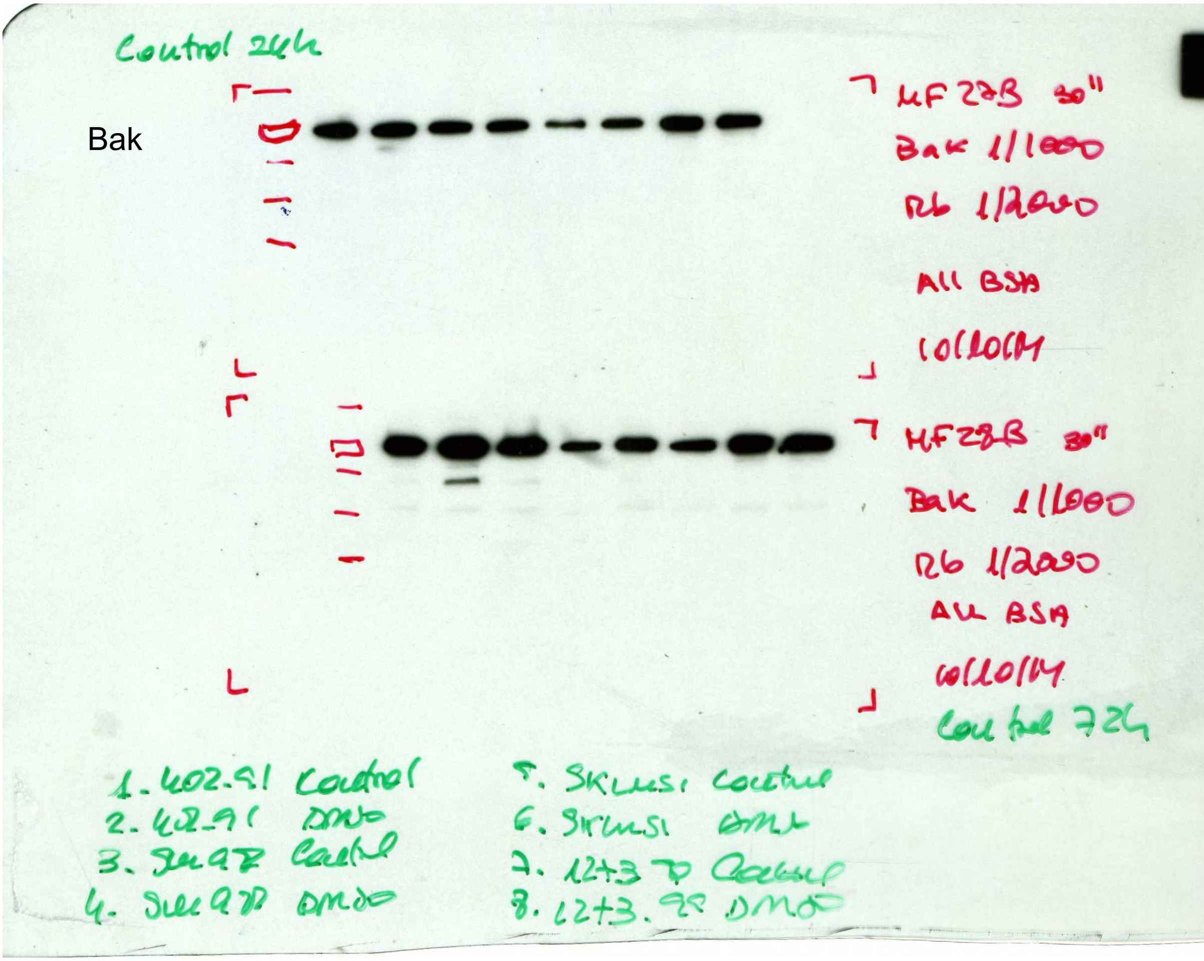

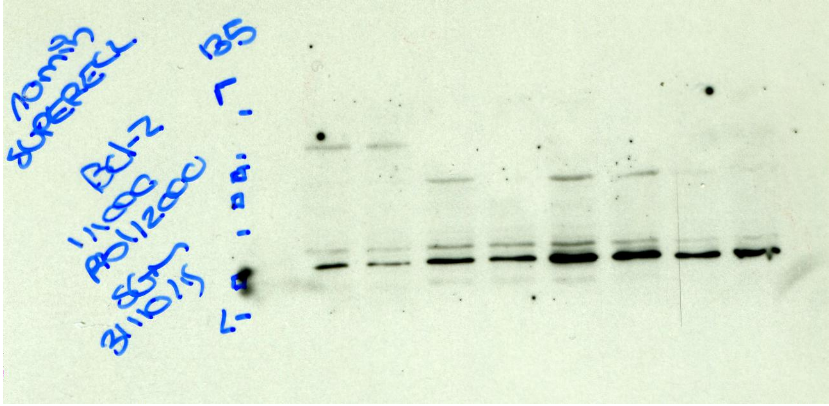

Rello-Varona *et al.*:

Bcl-x_L interferes in Dinaciclib-induced apoptosis in sarcomas.

Figure 3A

Bcl-x_L

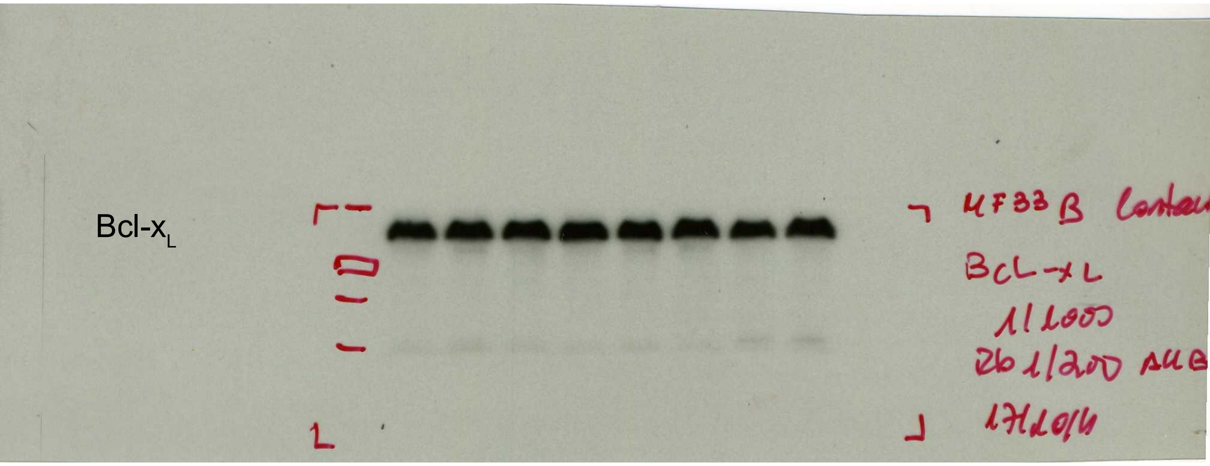

Mcl-1

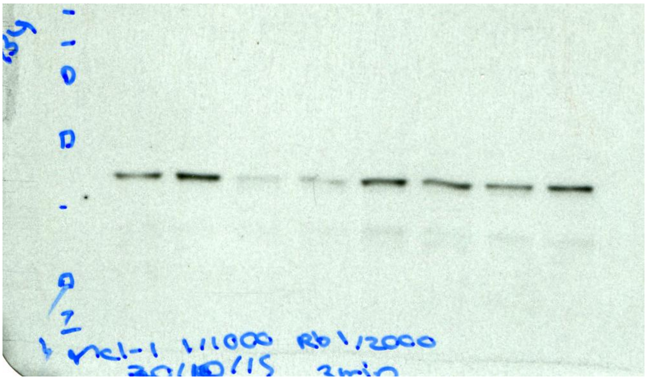

p53

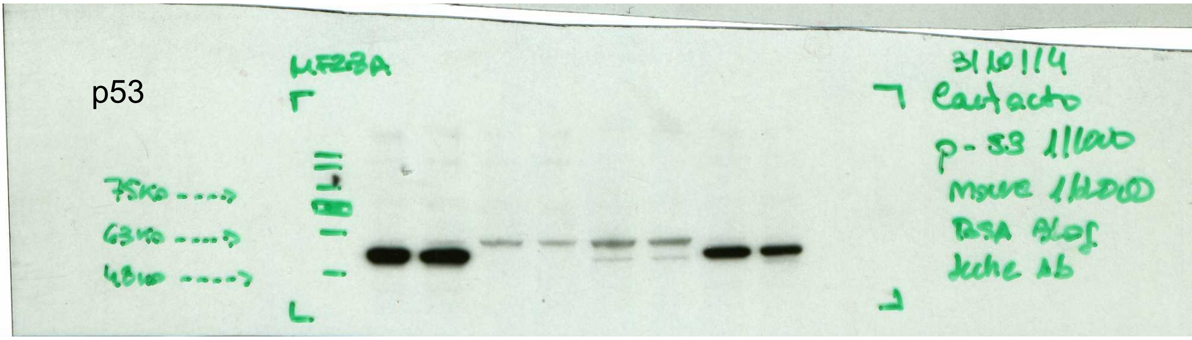

Rello-Varona *et al.*:

Bcl-x_L interferes in Dinaciclib-induced apoptosis in sarcomas.

Figure 3A

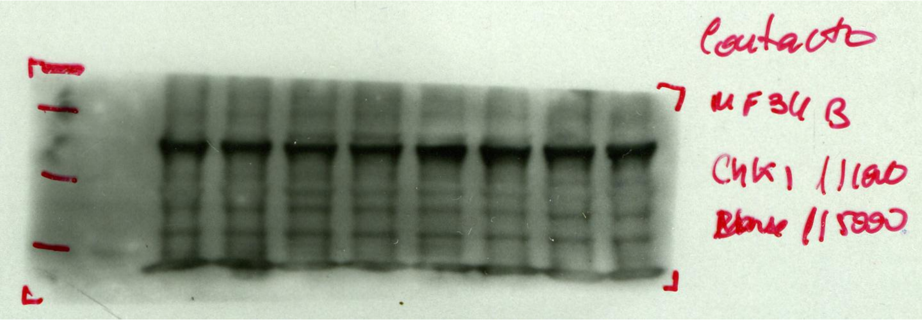

Chk-1

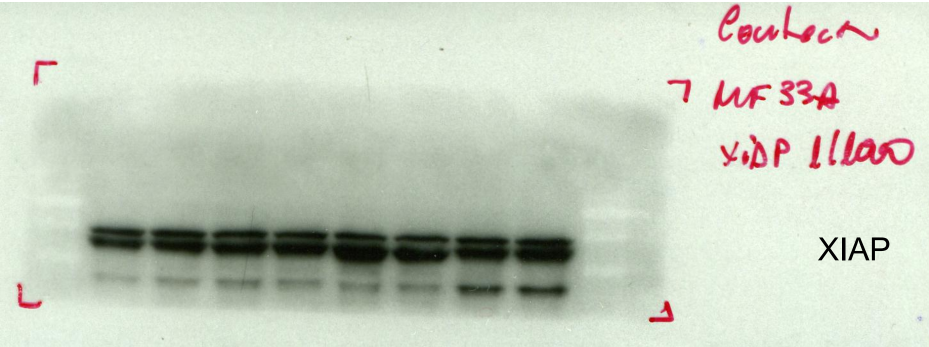

XIAP

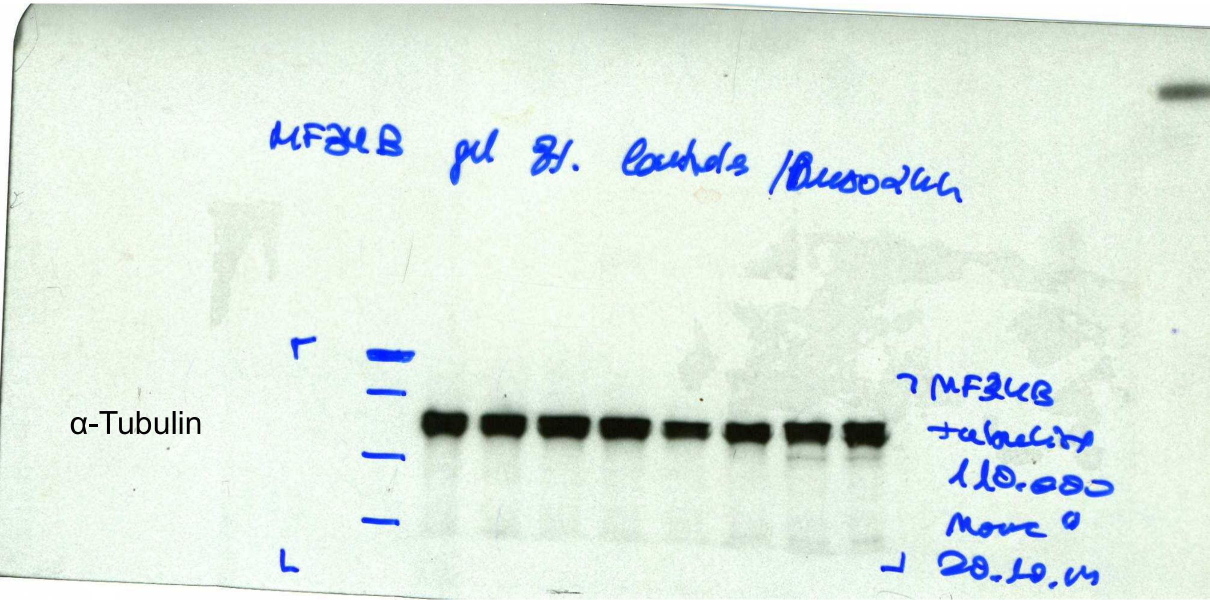

α-Tubulin

Rello-Varona *et al.*:

Bcl-x_L interferes in Dinaciclib-induced apoptosis in sarcomas.

Figure 3B

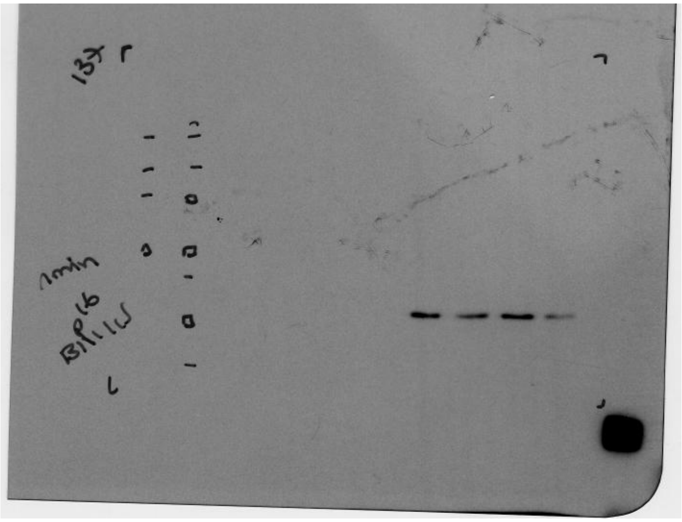

p16

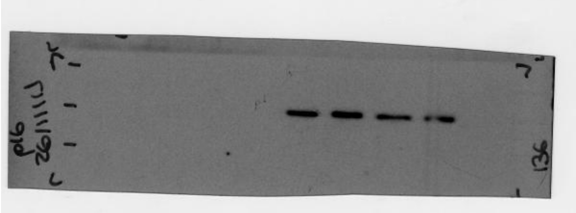

p16

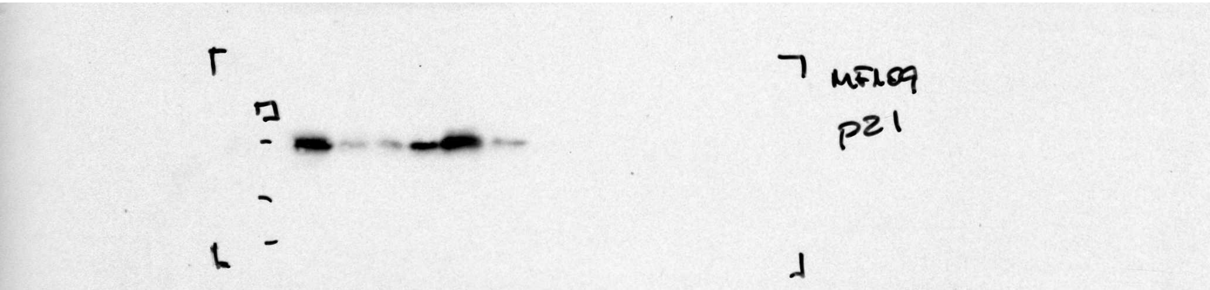

p21

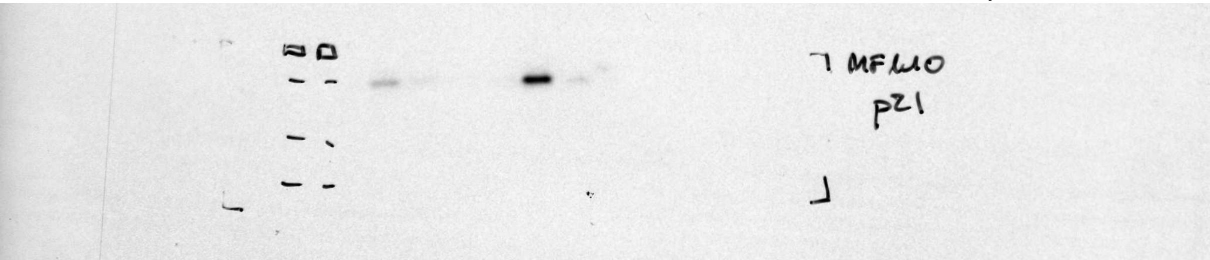

Rello-Varona *et al.*:

Bcl-x_L interferes in Dinaciclib-induced apoptosis in sarcomas.

Figure 3B

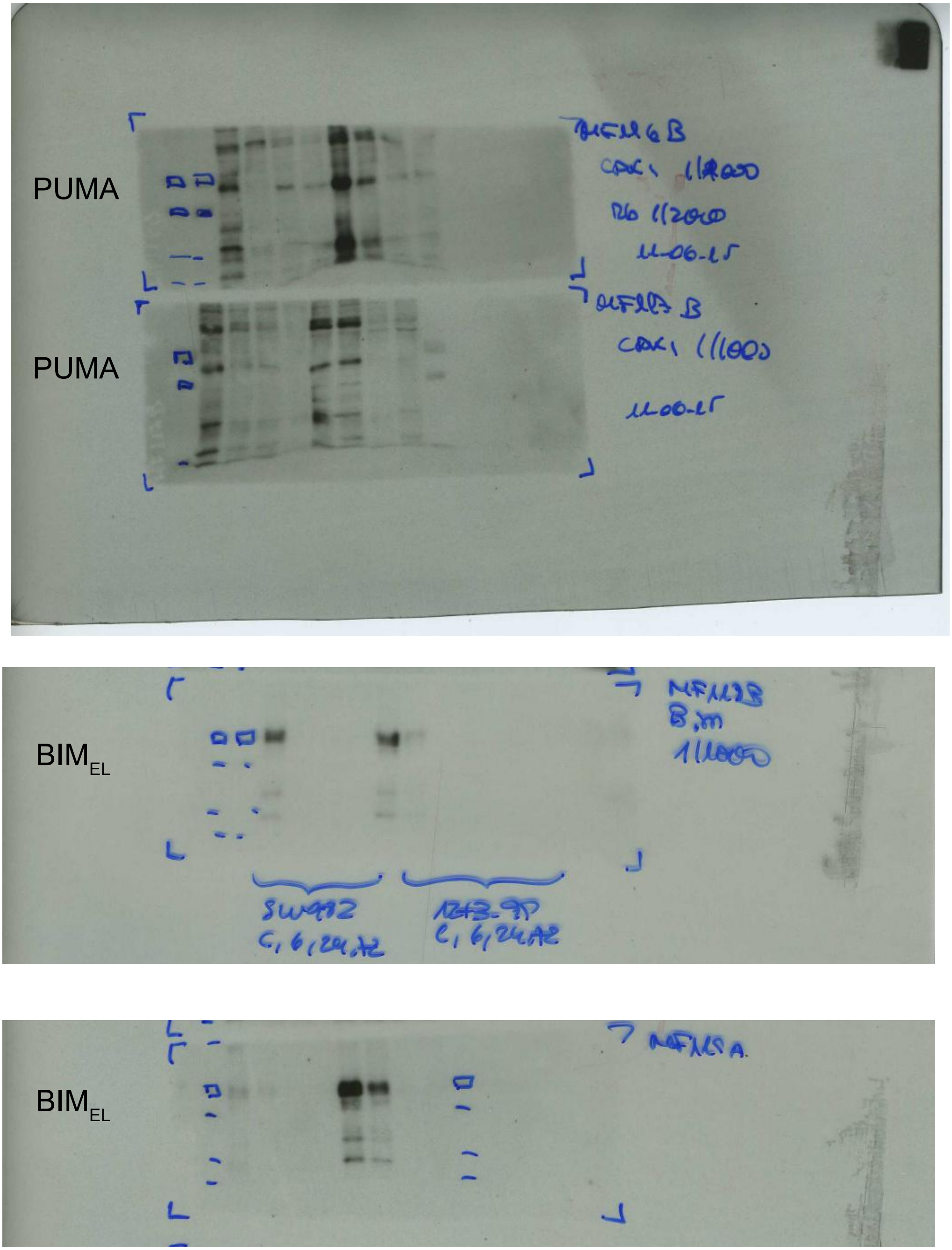

Rello-Varona *et al.*:

Bcl-x_L interferes in Dinaciclib-induced apoptosis in sarcomas.

Figure 3B

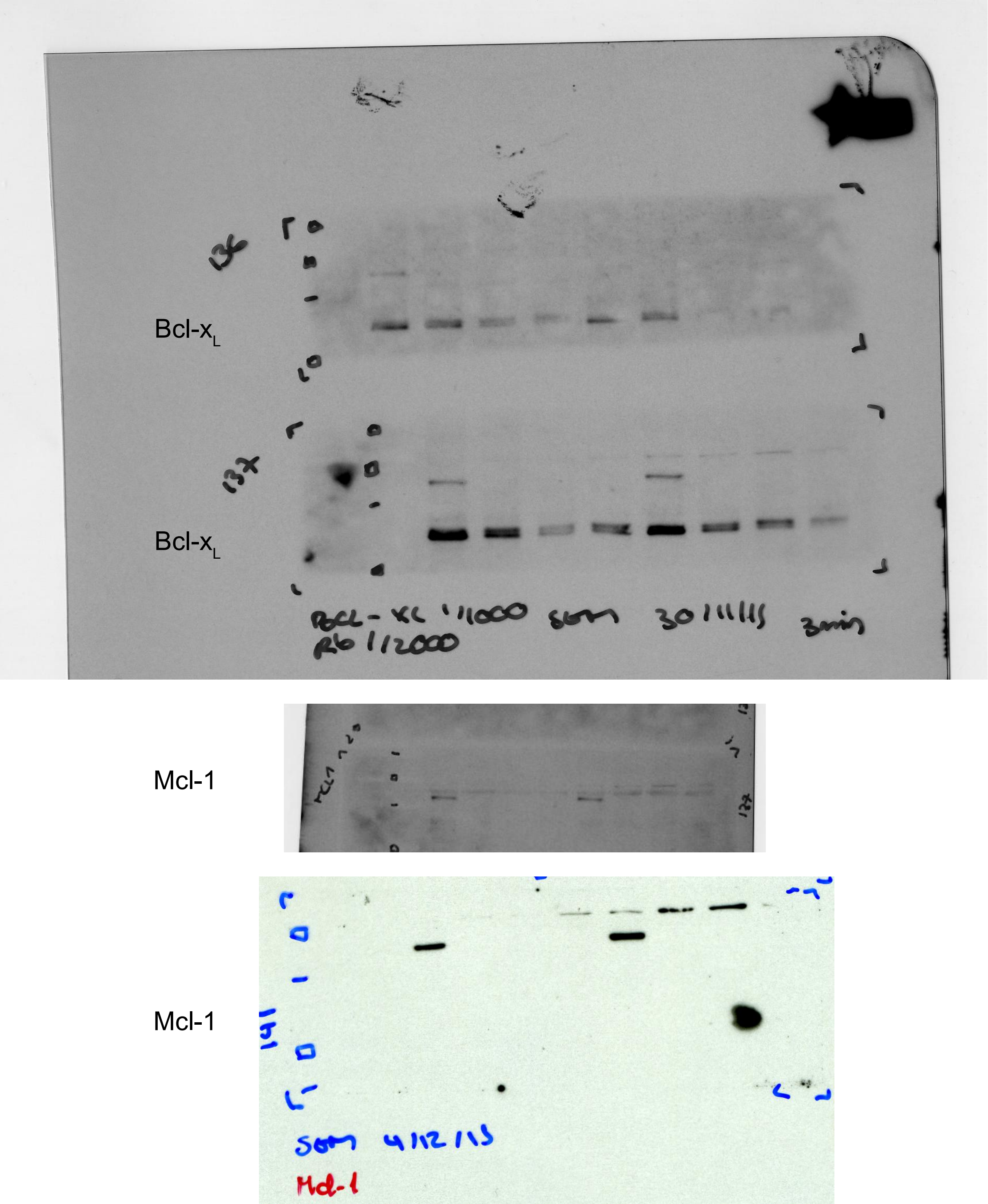

Rello-Varona *et al.*:

Bcl-x_L interferes in Dinaciclib-induced apoptosis in sarcomas.

Figure 3B

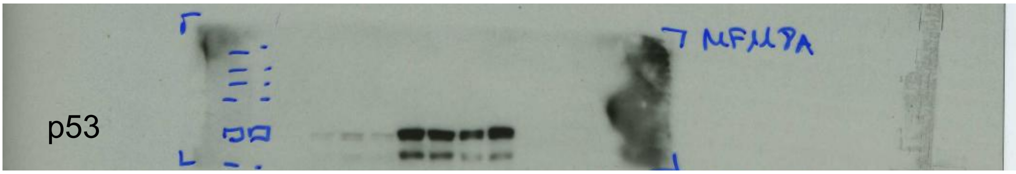

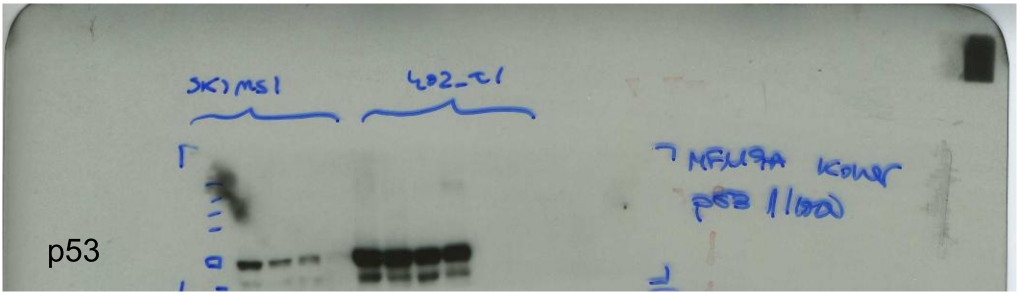

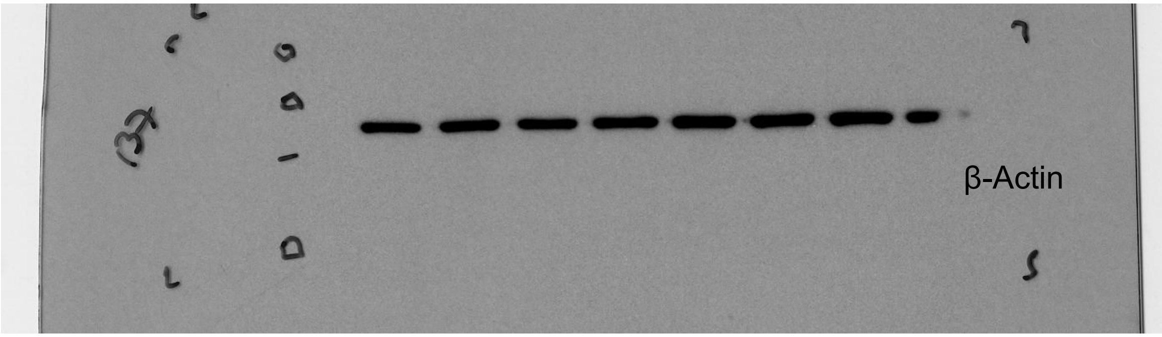

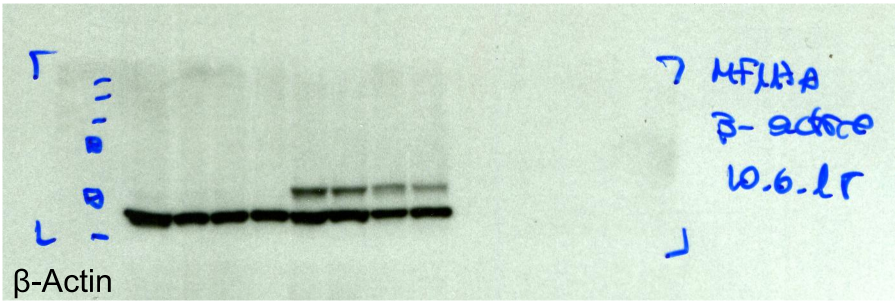

Rello-Varona *et al.*:
Bcl-x_L interferes in Dinaciclib-induced apoptosis in sarcomas.

Supplementary Figure 1C

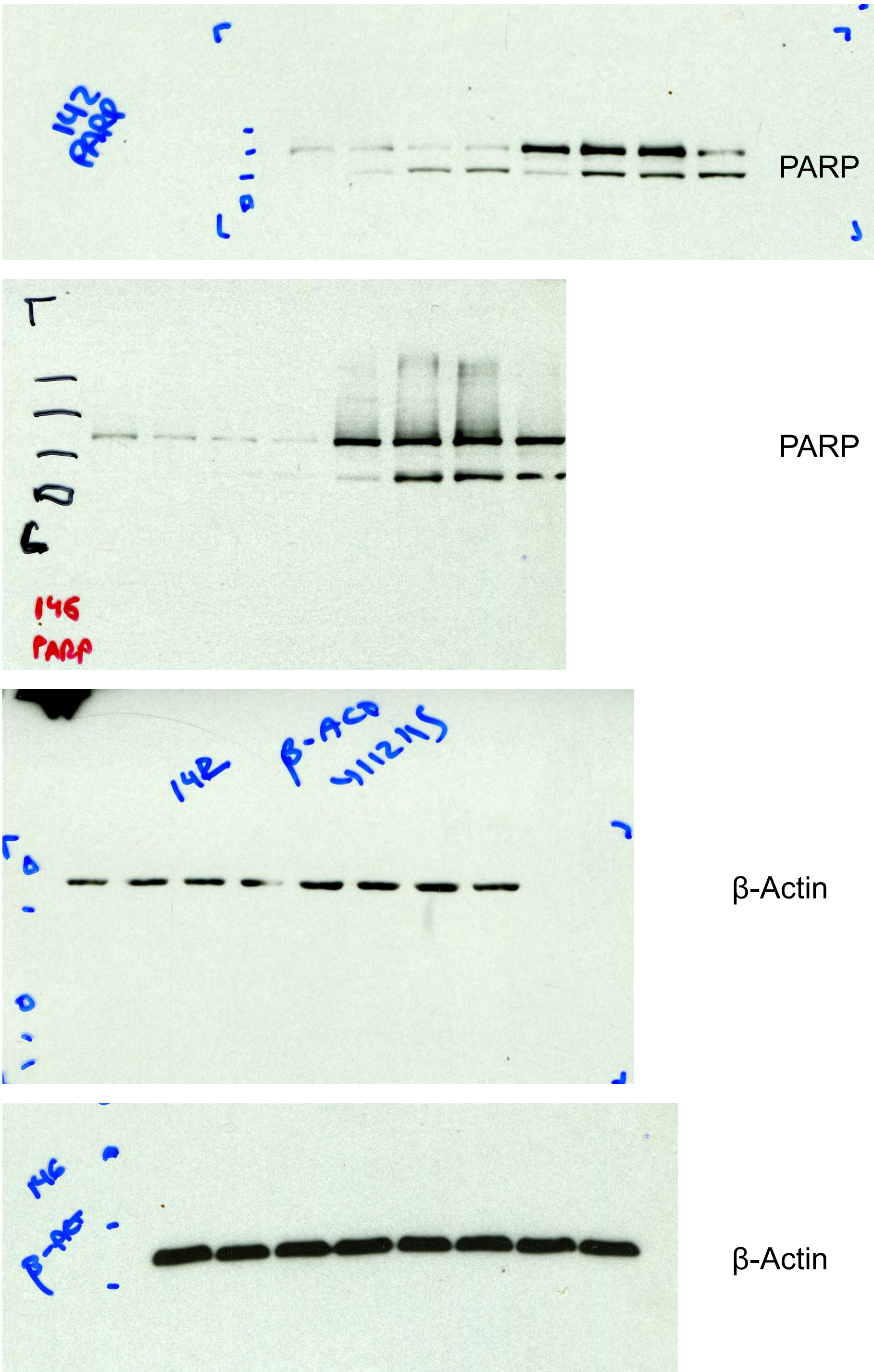

Rello-Varona *et al.*:

Bcl-x_L interferes in Dinaciclib-induced apoptosis in sarcomas.

Supplementary Figure 3B

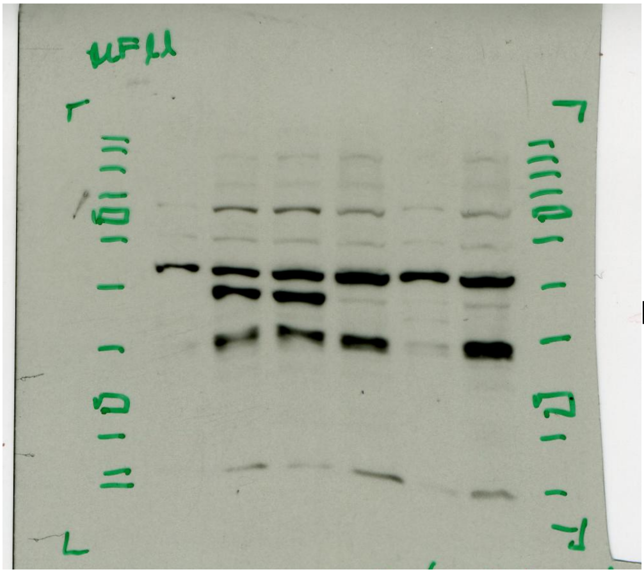

Mcl-1

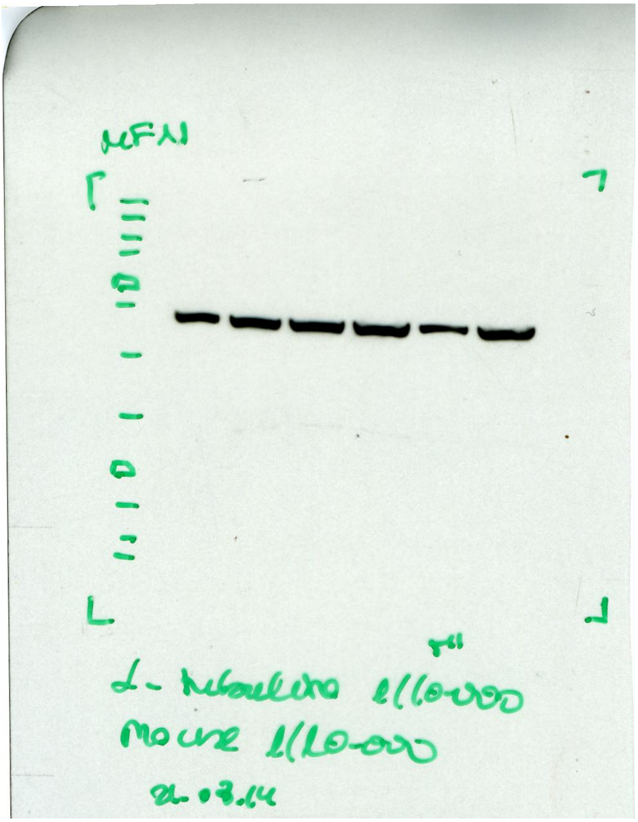

α-Tubulin

Rello-Varona *et al.*:

Bcl-x_L interferes in Dinaciclib-induced apoptosis in sarcomas.

Supplementary Figure 3D

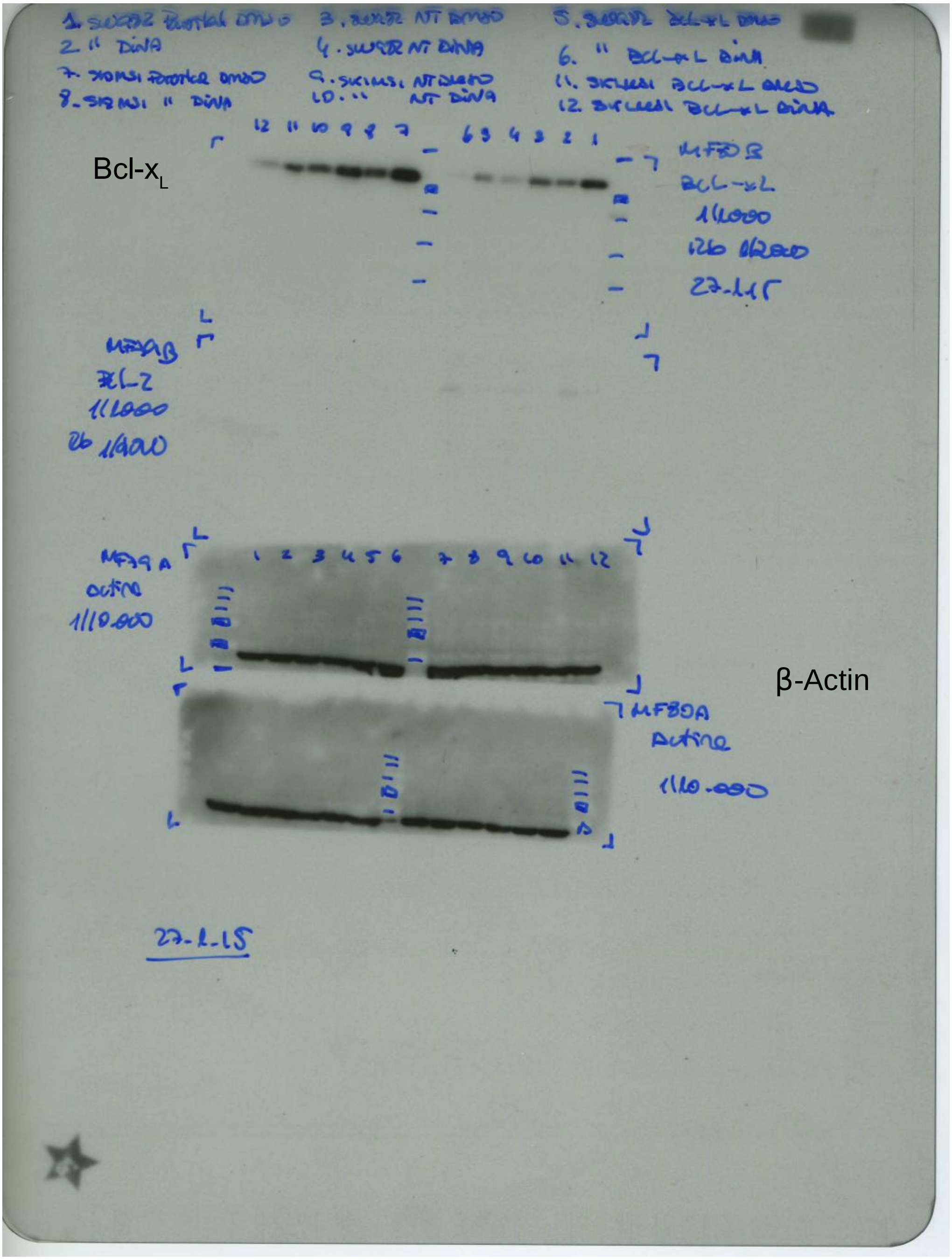

Supplement: Supplementary file 1 — Supplementary Information [file 41598_2019_40106_MOESM1_ESM.pdf]
